# The Public's Intended Uptake of Hypothetical Esophageal Adenocarcinoma Screening Scenarios: A Nationwide Survey

**DOI:** 10.14309/ajg.0000000000002812

**Published:** 2024-04-15

**Authors:** Jasmijn Sijben, Linda Rainey, Fleur Maas, Mireille J.M. Broeders, Peter D. Siersema, Yonne Peters

**Affiliations:** 1 Department of Gastroenterology and Hepatology, Radboud University Medical Center, Nijmegen, the Netherlands;; 2 Department for Health Evidence, Radboud University Medical Center, Nijmegen, the Netherlands;; 3 Dutch Expert Centre for Screening, Nijmegen, the Netherlands;; 4 Department of Gastroenterology and Hepatology, Erasmus MC - University Medical Center, Rotterdam, the Netherlands.

**Keywords:** Barrett's esophagus, esophageal adenocarcinoma, early cancer diagnosis, mass screening, patient-reported outcome, patient preference, patient participation

## Abstract

**INTRODUCTION:**

Screening for early esophageal adenocarcinoma (EAC) may potentially reduce EAC-related mortality and morbidity. This study aimed to examine the Dutch population's intended uptake of 3 hypothetical EAC screening test scenarios and preferences for potential future organization.

**METHODS:**

A total of 8,350 Dutch individuals aged 45–75 years were invited, of whom 2,258 completed a web-based survey. Participants were randomly assigned to 1 of 3 hypothetical screening test scenarios (i.e., transnasal endoscopy, ingestible cell collection device, or breath analysis). The primary outcome was intended uptake. Secondary outcomes included acceptance of screening eligibility criteria and preferences regarding invitation, counseling, and diagnostic follow-up. We performed exploratory univariable and multivariable regression analyses to assess which determinants were associated with EAC screening intent.

**RESULTS:**

Intended uptake of screening was highest in the breath analysis scenario (95%), followed by conventional upper endoscopy (78%), an ingestible cell collection device (75%), and transnasal endoscopy (68%) (*P* < 0.001). Anticipating discomfort was most strongly associated with decreased intention to undergo transnasal endoscopy (odds ratio 0.18, 95% confidence interval 0.11–0.29) or swallow a cell collection device (odds ratio 0.20, 95% confidence interval 0.13–0.32). Cancer worry and high acceptance of test sensitivity/specificity were consistently associated with a positive intention to participate in screening. Inviting persons for screening based on gastroesophageal reflux disease symptoms, age, or the output of a risk prediction model was acceptable to 74%, 69%, and 66%, respectively. Inviting only men was acceptable for only 41% of women. The majority (58%) preferred to be invited by a public health organization, and 32% of the participants preferred to discuss their decision to participate with a healthcare professional.

**DISCUSSION:**

Participants in this study self-selected through a web-based survey, potentially introducing selection bias. Participants generally intended to participate in EAC screening, although the level of intent depended on the discomfort and performance associated with the offered screening test. Determining eligibility based on gastroesophageal reflux disease symptoms, age, or a risk calculator, but not sex, would be acceptable to most individuals.

## INTRODUCTION

The incidence of esophageal adenocarcinoma (EAC) has increased substantially in Western countries (1). More than 40% of patients with EAC are diagnosed at a metastasized stage and consequently do not benefit from potentially curative treatments (2). This has unlocked great international interest in screening for EAC and its precursor Barrett's esophagus (BE). Current gastroenterology guidelines suggest screening for BE in a selected higher risk population (3–5). The higher risk population is defined as individuals with gastroesophageal reflux disease (GERD) combined with other risk factors such as age older than 50 years, male sex, White race, obesity, and family history, although the precise definition of risk factors varies among guidelines (3–5). Persons with confirmed BE are followed up with periodic endoscopic surveillance and are offered endoscopic therapy when dysplasia or early EAC is detected. This set of interventions may theoretically prevent the development of dysplasia into EAC, induce a shift toward earlier stage EAC at diagnosis, increase the opportunity for curative treatment, and improve survival. To date, evidence supporting the effectiveness of EAC screening is limited to modeling studies (6). Substantial research efforts are being dedicated to optimizing screening policy. Aligning screening policy with individuals' preferences and their personal drivers and barriers to attend screening could improve uptake and thereby potentially the effectiveness of screening.

Previous screening trials and surveys performed in the community suggest that the choice of the screening test will likely affect individuals' willingness to participate (7–11). The public has been shown to prefer noninvasive technology, such as a breath analysis test, but only if it is very accurate (9,10). Ingestible cell collection devices, such as the Cytosponge-TFF3 technology, are well accepted and cause little anxiety among participants in screening trials (12–14). However, screening uptake in these trials was suboptimal, possibly because individuals who declined the invitation were deterred by anticipated discomfort associated with this test (15). Another test option, transnasal endoscopy, was preferred over conventional upper endoscopy (esophagogastroduodenoscopy [EGD]) with higher uptake in screening trials (7,9,11). Despite this growing body of evidence, it remains unclear how the public would perceive a multitier screening process in which one of these minimally invasive screening tests is combined with confirmatory endoscopy.

Public acceptance of the screening policy may also be affected by the methods used for selecting a higher risk population. Risk factors of EAC may be used either alone or combined in risk prediction models to determine screening eligibility. A first qualitative exploration revealed that sex and race-specific screening eligibility may result in fear of exclusion, and the introduction of waist circumference and smoking as eligibility criteria could be perceived as stigmatizing (15). Individuals further preferred to discuss risk-based screening policies with a healthcare professional, which would have implications for healthcare resources. However, these findings have not been confirmed in larger study samples.

To address these knowledge gaps, this study will assess the public's intended uptake of different EAC screening scenarios, determinants associated with intended uptake, and preferences for its organization.

## METHODS

### Study design

Cross-sectional data were collected in the Netherlands between February and June 2023 using a web-based survey. Ethics approval was acquired from the regional ethics committee CMO Arnhem-Nijmegen in the Netherlands (ref no. 2023-16163). Informed consent was obtained online before the start of the survey. The protocol for this study has been registered in ClinicalTrials.gov (NCT05690958). The survey was conducted and is reported in accordance with the Checklist for Reporting Results of Internet E-Surveys (CHERRIES) (16).

### Definitions

In this article, screening is defined as offering a medical examination (i.e., EGD, transnasal endoscopy, ingestible cell collection device, or breath analysis) aimed at detection of BE and/or BE-related neoplasia, followed by surveillance of BE and treatment of detected dysplasia and EAC. Since the ultimate goal of this strategy is to detect and treat cancer, we refer to this definition as EAC screening, thus including screening for BE.

### Participants and recruitment

A total of 8,350 individuals, aged 45–75 years, were randomly sampled from 6 Dutch municipal population registries that were diverse in terms of geographical region, average socioeconomic status, and degree of urbanization (see Supplementary Table S1 and Figure S1, Supplementary Digital Contents 1, http://links.lww.com/AJG/D250, and 2, http://links.lww.com/AJG/D251, for detailed information). In the absence of established age cutoffs for screening in current guidelines, the lower age limit was determined based on a recent modeling study (17), while the upper limit adhered to BE surveillance age cutoffs to encompass all individuals potentially eligible for EAC screening. Participants were eligible to participate in the study if they understood Dutch language and had internet connection using a cellphone, tablet, or computer. We did not prevent participants with a history of esophageal cancer from completing the survey, but their data were subsequently excluded from analyses. Selected individuals were sent an invitation letter through postal mail containing a URL and corresponding QR code to the survey, which were linked to a unique participant identification number to prevent duplicate entries. No financial incentives were offered either upon registration or after survey completion.

### Survey

**Figure 1.**
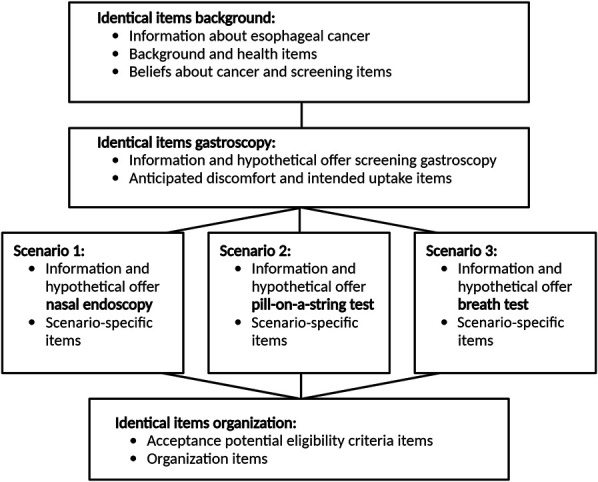
Schematic design of the survey.

The construction of the survey was based on a systematic literature review and qualitative focus group data (15,18). Survey items were adapted from existing measures and studies, reviewed by the study team, piloted in cognitive interviews (n = 7), and tested for technical functionality in Castor EDC (electronic data capture) (19) (n = 3) before this study. Validated questions were used whenever possible. Three versions of the survey were generated in Castor EDC, all of which were identical, except for the hypothetical screening test scenario (Figure 1 shows the schematic design of the survey). Individuals selected by the municipalities were randomly assigned to 1 of the 3 survey versions before the distribution of invitations. Full details on the source, wording, and coding of survey items are available in Supplementary Table S2 (see Supplementary Digital Content 3, http://links.lww.com/AJG/D252). In brief, the survey started with information about the development of esophageal cancer. Participants then provided data about potential determinants of screening uptake, including their sociodemographic background (i.e., age, sex, education level, civil status, and ethnicity), familiarity with the topic (i.e., upper endoscopy experience, current GERD symptoms, personal history of cancer, prior screening behavior, and knowing someone affected by esophageal cancer), and psychological variables (i.e., general cancer worry, perceived risk of esophageal cancer, and beliefs about cancer screening). Next, participants were presented with the hypothetical screening test scenario they were assigned to (i.e., transnasal endoscopy, ingestible cell collection device, or breath analysis). Each scenario contained information about the assigned test, followed by Likert scale items measuring acceptance of the sensitivity and specificity of the assigned test, anticipated discomfort and gagging, and intended participation. In addition, the assigned test was hypothetically offered in combination with confirmatory EGD only in the event of a positive result, to evaluate the acceptability of a 2-step screening policy. Next, we informed participants about a series of potential approaches to determining eligibility for screening. We used Likert scales to determine if these (i.e., based on age or GERD, inviting only men, or using a risk prediction model) would be acceptable and if participants would be willing to provide the required information (i.e., on skin color, smoking, waist circumference, family history, and access to their medical file) and a blood sample. The survey ended with questions about the perceived influence of test location, whether screening was recommended by their general practitioner (GP), out-of-pocket costs, reminders, and having a busy lifestyle on intended participation in screening and participants' views on the organization of screening (i.e., preferred invitation method, perceived need for consultation and public education campaigns, and acceptability of using public resources for screening).

### Sample size

**Figure 2.**
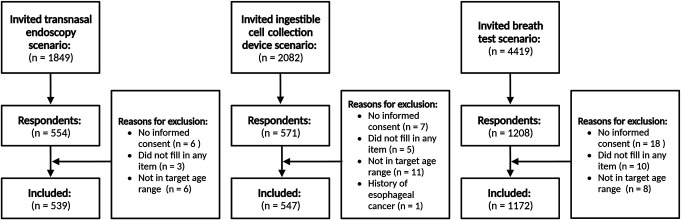
Study flow. Larger number of individuals invited for the breath test scenario due to the sample size calculation (see Methods).

To determine the sample size for a binomial logistic regression analysis, we used a previous study to estimate the probability of the primary outcome (intended participation) in each screening scenario (7). This probability was multiplied by 190 (19 potential determinants x 10 events per variable) for each scenario, resulting in required sample sizes of 462 (transnasal endoscopy scenario), 521 (ingestible cell collection device scenario), and 1,105 (breath test scenario). Based on an assumed participation rate of 25%, 8,350 invitations were sent in total (Figure 2).

### Analysis

Descriptive statistics are presented to establish participants' general characteristics and study outcomes. These were further stratified by the assigned hypothetical screening test scenario (i.e., transnasal endoscopy, ingestible cell collection device, and breath test).

Univariable and multivariable logistic regression analyses were performed to explore the association between intended participation in each screening test scenario and a set of potential determinants (i.e., age, sex, education, civil status, GERD symptoms, upper endoscopy experience, personal history of cancer, knowing someone affected by esophageal cancer, cancer worry, perceived esophageal cancer risk, prior participation in cancer screening programs, beliefs about cancer screening, anticipated discomfort, and acceptability of test performance). The chosen determinants were based on a systematic review of the literature and our previous focus group study (13,15). For these analyses, missing data were handled using pairwise deletion (not exceeding 3.3%). Determinants with a *P*-value of <0.2 in univariable analyses were included in the multivariable model with backward selection (20). In the breath analysis scenario, the sample sizes for most variables were too small to perform logistic regression analyses. Multivariable analyses were, therefore, not performed. This was due to the higher number of individuals with positive intent to participate (95%) than assumed based on the prior literature (83%) in the sample size calculation. To explore the potential impact of nonresponder bias, we performed sensitivity analyses of subsamples that closely represented the characteristics of the general population (details in Supplementary Methods, Supplementary Digital Content 4, http://links.lww.com/AJG/D253). Statistical analyses were conducted using SPSS version 27 (IBM, Armonk, NY). A 2-tailed *P* value <0.05 was considered significant.

## RESULTS

### Characteristics and representativeness of the study sample

Of the 8,350 individuals invited, 2,258 were included (response rate: 27.0%, stratified by municipality in Supplementary Table S1, Supplementary Digital Content 1, http://links.lww.com/AJG/D250). Figure 2 shows that 75 individuals were excluded (31 did not provide informed consent, 18 did not answer any questions, 25 were not in the target age group, and 1 had a history of esophageal cancer). The mean age of the final sample was 60.5 years (SD 8.3), 50% were female, and there was a good representation of different education levels (Table 1). Most were married (71%) and had a White ethnic background (96%). The mean score on the reflux disease questionnaire was 0.3 (SD 0.7). Knowing someone affected by esophageal cancer was commonly reported (40%). Most participants estimated their esophageal cancer risk to be average (69%) or below average (24%). Participants generally had positive beliefs about cancer screening and reported high previous participation in population-based cancer screening programs (89%). Participants' characteristics did not differ based on the assigned screening test scenario, except for upper endoscopy experience which was less prevalent in the ingestible cell collection device scenario (18% vs 23% in the transnasal endoscopy and 24% in the breath test scenario, *P* = 0.02).

**Table 1.**
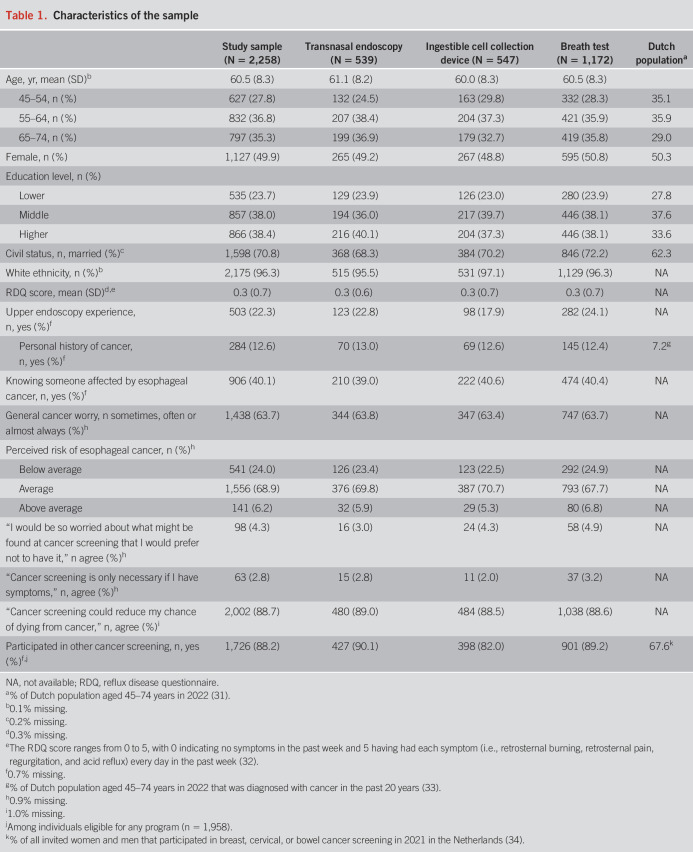
Characteristics of the sample

### Intended participation and screening preferences

As summarized in Table 2, most participants (78%) stated that they would absolutely or likely undergo EAC screening with EGD. Intended participation in the novel tests was highest in the breath test scenario (95%), followed by the ingestible cell collection device (75%) and transnasal endoscopy (68%, *P* < 0.001). The sensitivity analysis conducted on a subsample (n = 683) matching the general populations' proportion of prior cancer screening participation showed minor deviations: 74% expressed an intention to undergo screening with EGD, 93% with the breath test, 71% with an ingestible cell collection device, and 64% with transnasal endoscopy (see Supplementary Methods, Supplementary Digital Content 4, http://links.lww.com/AJG/D253). Acceptability of undergoing 2 tests to receive a final result decreased with test invasiveness: 3% in the breath test scenario found this unacceptable, 9% in the ingestible cell collection device scenario, and 13% in the transnasal endoscopy scenario.

**Table 2.**
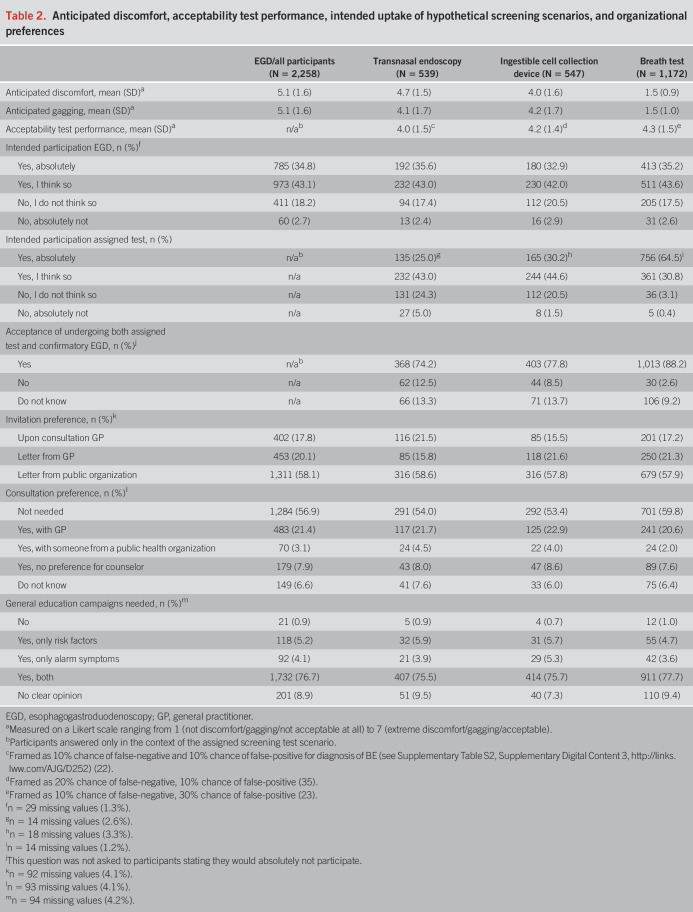
Anticipated discomfort, acceptability test performance, intended uptake of hypothetical screening scenarios, and organizational preferences

The mean anticipated discomfort score (1 = no discomfort and 7 = extreme discomfort) was lowest for the breath test (1.5, SD 0.9), followed by an ingestible cell collection device (4.0, SD 1.6) and transnasal endoscopy (4.7, SD 1.5). Acceptability scores of the diagnostic performance of each test were comparable (Table 2).

Participants generally preferred to receive the screening invitation in a letter (78%). One-third wished to discuss the screening offer with a healthcare professional, preferably with their GP. Invitation and consultation preferences were comparable among the various screening test scenarios.

### Acceptability of eligibility criteria

**Figure 3.**
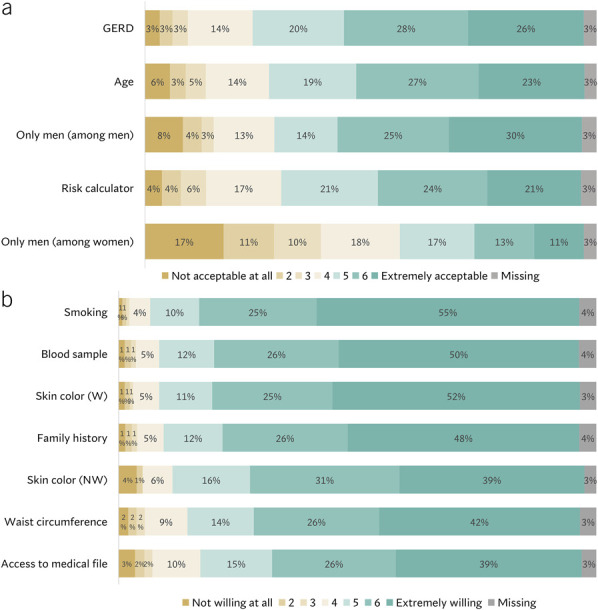
(**a**) Participants' acceptance of using 4 potential eligibility criteria to select people for screening. Measured on a Likert scale ranging from 1 (not acceptable at all) to 7 (extremely acceptable), with a score of ≥5 considered as accepting. (**b**) Participants' willingness to provide access to their medical file; a blood sample; or information about their smoking status, skin color, family history, and waist circumference as input for a risk calculator. Measured on a Likert scale ranging from 1 (not willing at all) to 7 (extremely willing), with a score of ≥5 considered as willing to provide. Data on willingness to provide skin color is reported separately for White (W) and non-White (NW) individuals. GERD, gastroesophageal reflux disease.

Figure 3a shows acceptability scores for using personal characteristics to determine screening eligibility. Overall, the majority responded that it was acceptable to use GERD (74%); age (69%); or a risk prediction model incorporating age, sex, GERD symptoms, waist circumference, smoking habits, skin color, and family history (66%). Among men, 69% responded that it was acceptable to invite only men, whereas only 41% of participating women thought this was acceptable.

Most participants (80%) were willing to provide access to their medical file; give a blood sample (88%); or disclose their smoking status (90%), waist circumference (82%), skin color (86% among non-Whites, 88% among Whites), or family history (86%) as input for a risk calculator (Figure 3b).

### Determinants associated with intended participation

Table 3 presents the results of the explorative analyses into determinants associated with EAC screening intent. Anticipated discomfort was most strongly associated with decreased intent to undergo transnasal endoscopy (odds ratio [OR] 0.18, 95% confidence interval [CI] 0.11–0.29) or swallow a cell collection device (OR 0.20, 95% CI 0.13–0.32). High acceptance of the risk of a false-positive or false-negative result was associated with increased intent to participate (transnasal endoscopy: OR 3.16, 95% CI 1.99–5.01; ingestible cell collection device: OR 1.80, 95% CI 1.12–2.88). Cancer worry was associated with increased intended uptake (transnasal endoscopy: OR 2.10, 95% CI 1.37–3.23; ingestible cell collection device: OR 2.03, 95% CI 1.28–3.22), similar to the perceived personal risk of esophageal cancer (average vs below average OR 2.05, 95% CI 1.23–3.40). Endorsing negative beliefs about cancer screening was associated with decreased intent to participate in screening (Table 3). Higher age was marginally associated with decreased interest in screening.

**Table 3.**
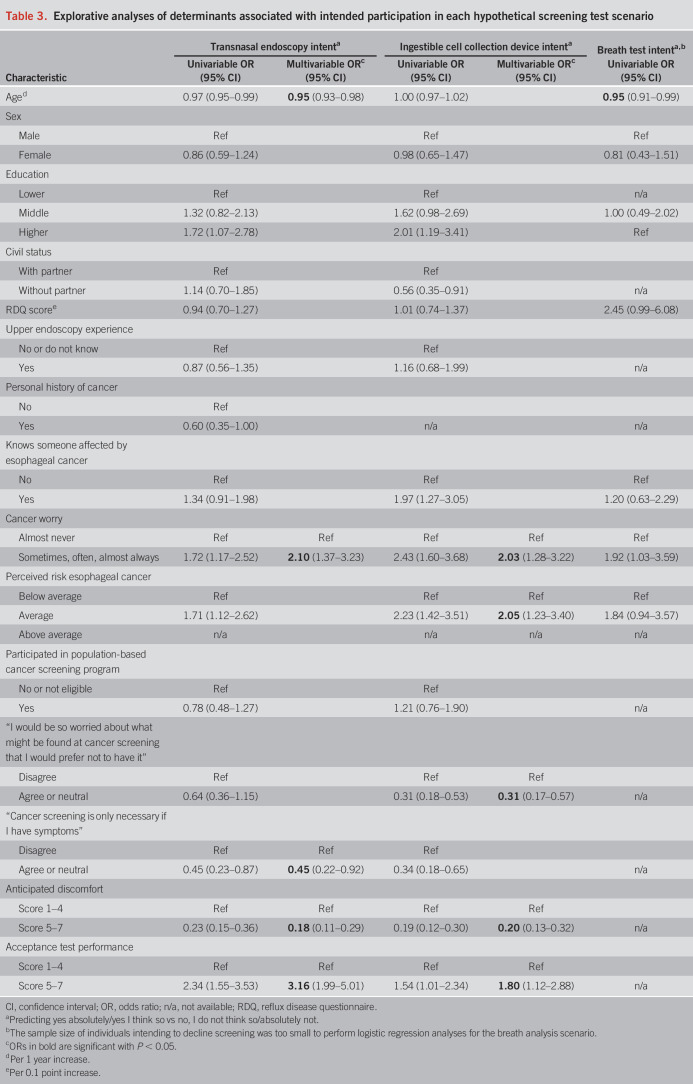
Explorative analyses of determinants associated with intended participation in each hypothetical screening test scenario

**Figure 4.**
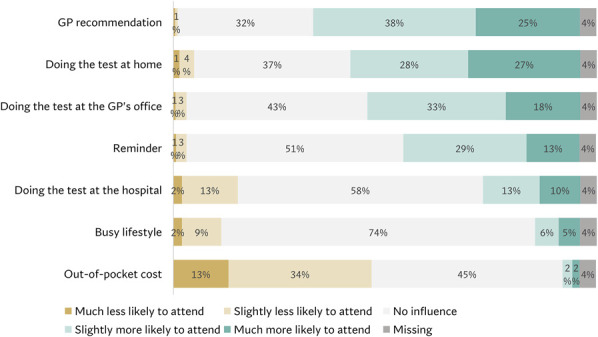
Perceived influence of external factors on intended participation in hypothetical EAC screening. EAC, esophageal adenocarcinoma; GP, general practitioner.

The perceived influence of other external factors on intent to participate in EAC screening is reported in Figure 4. Recommendation by the GP, being able to do the test at home or at the GP's office, and need to pay out-of-pocket costs were the most influential factors.

## DISCUSSION

This study shows that the choice of screening test significantly contributes to a person's intent to participate in EAC screening, with particular support for the breath test. Anticipated discomfort associated with the test and acceptance of the test's sensitivity/specificity were most strongly associated with intended uptake. Individuals also showed higher screening intent if they reported higher general cancer worry, disagreed with pessimistic beliefs about cancer screening, if their GP recommended it, and if they could do the test at home or at the GPs office. Using a risk prediction model was just as well-received as using age or GERD alone as the selection criterion.

Fear of discomfort of the screening test and the need to pay out-of-pocket costs are barriers to EAC screening uptake, which is consistent with a recent survey conducted in the United States (21). Furthermore, the observed differences in intended uptake among screening tests are in line with the prior literature, which consistently reported that individuals prefer noninvasive tests that are easily accessible and cause no pain or esophageal damage (9,10,15). Remarkably, participants reported comparable levels of acceptability regarding test accuracy for both transnasal endoscopy (with a sensitivity and specificity of 90%) (22) and breath analysis (with a sensitivity of 90% and specificity of 70%) (23). This finding mirrors previous discrete choice experiments in which test sensitivity particularly affected screening intent (9,24,25), which may be driven by individuals' fear of missing cancer (15). It should be kept in mind that the preference for the breath test can significantly shift if the test performs less well in validation studies since individuals highly value test accuracy (9,10,15). Another limitation of the breath test is its current unavailability for clinical use and the absence of a recommendation in guidelines. This contrasts with transnasal endoscopy and ingestible cell collection devices, which are suggested alternatives to EGD according to the recent American College of Gastroenterology guideline (3).

This study further found that transnasal endoscopy was inferior to EGD in terms of intended uptake, which contrasts with previous screening studies (7,9,11). This incongruity can likely be explained by the fact that participants in this study were made aware that transnasal endoscopy has an approximately 10% risk of being false-negative or false-positive because it may yield unreliable pathology samples due to the small size of the biopsy forceps, necessitating conventional EGD when endoscopic abnormalities are observed (22). The need to undergo 2 tests for a final result is less acceptable if both tests are considered uncomfortable, which may lead individuals to be more inclined to undergo EGD directly (15).

This study's explorative evaluation of risk stratification within esophageal cancer screening conveys a positive first impression of the acceptability of using a risk prediction model, which was considered as acceptable as using age alone. Among female participants, selecting only male individuals for EAC screening was less accepted than all other eligibility scenarios, which is in line with our prior focus group study (15). This finding also aligns with a survey eliciting risk stratification preferences for kidney cancer screening, a cancer with a male predominance pattern similar to esophageal cancer, which also reported up to half of women were uncomfortable with using sex as the selection criterion (26). Furthermore, our findings are consistent with general public support for risk stratification within population-based cancer screening, as reported in a recent systematic review (27). This review also reported individuals' concerns regarding risk-stratified screening, such as data security, the propensity of the policy to be unfair, the negative emotions associated with receiving a personalized risk estimate, and individuals' worry that risk prediction is insufficiently linked to actual cancer development (27). Another systematic review showed risk-stratified cancer screening is largely acceptable to healthcare professionals, but a strong evidence base and support and training are required to successfully facilitate implementation (28). These implications should also be considered during potential future incorporation of risk prediction models in EAC screening policies.

Around 32% of the participants wanted a risk consultation, with a clear preference for their GP over a public health organization employee to do this. This aligns with our previous qualitative study, in which GP involvement was also preferred based on trust, familiarity, convenience, and a personal approach (15). The feasibility of EAC risk assessment in primary care has not been investigated, but pilots of breast cancer risk assessment and counseling in primary care settings highlighted significant barriers. The impact on workflow, consultation time, and GPs' limited knowledge about interpretation of cancer risk and screening guidelines raise doubts about the feasibility of assigning this task to GPs (29). Instead, public health organizations might perform communication activities, potentially supported by shared decision aids or artificial intelligence-powered chatbots.

This study benefited from a large, population-based sample with a balanced representation of age groups, men and women, and education levels. Randomizing participants between hypothetical screening scenarios allowed us to estimate the intended uptake of 3 potential screening tests without overloading each participant with information and questions. Participants in the 3 independent randomization groups expressed similar levels of intent to undergo EGD for EAC screening, indicating that baseline screening interest was comparable in each group. There are, however, some limitations that need to be considered when interpreting the results. First, the 27% response rate introduces potential self-selection biases, including volunteer bias (i.e., more motivated or health-conscious individuals filling out the survey), selection bias related to the web-based design (i.e., individuals more comfortable with technology filling out the survey), and nonresponder bias (i.e., characteristics of individuals who choose not to fill out the survey differ systematically from those who do). However, sensitivity analyses of subsamples matching general population characteristics yielded comparable results, suggesting a limited impact of nonresponder bias. Second, the selection of screening tests was primarily informed by European norms, where endoscopy (i.e., EGD in this case) is not commonly used for widespread population screening. However, we provided data on anticipated discomfort, gagging, and the intended uptake of EGD, allowing for comparisons with data from other screening tests. Third, the exposure to a single screening test may limit generalizability to real-world decision-making scenarios where individuals may need to weigh the pros and cons of multiple options. In addition, the weak correlation between intent and behavior (30) suggests actual uptake of a real-world screening policy is likely lower than the estimates of intent presented here. The measurement of screening intent is mainly useful to explore relative interest in screening scenarios.

A risk-based esophageal cancer screening policy would be well-received in principle. Our findings highlight the influence of the offered screening test on intended uptake, with particular support for the breath test. Based on these results, we recommend focusing research on achieving high sensitivity and specificity of noninvasive screening methods in primary care populations and managing individuals' expectations of the discomfort and risks associated with ingestible cell collection devices. Our findings suggest that the public perceives transnasal endoscopy combined with confirmatory endoscopy as the least favorable test option. If research in this area is pursued, we recommend improving dysplasia detection with transnasal endoscopy to obviate the need for confirmatory EGD. Selecting the target screening population based on age, GERD symptoms, or a risk calculator would be acceptable to most individuals. Using sex alone to determine screening eligibility does not appear to receive broad public support. Further work is now needed to improve risk prediction models and explore the willingness and capacity of primary care and public health organizations to take up EAC screening activities.

## CONFLICTS OF INTEREST

**Guarantor of the article:** Peter D. Siersema, MD, FACG.

**Specific author contributions:** L.R., M.J.M.B., Y.P., and P.D.S. contributed to the design of the study and measures, data interpretation, project supervision, and writing of the manuscript. F.M. contributed to project administration and statistical analysis. J.S. contributed to the design of the study and measures, project administration, data interpretation, statistical analysis, visualization, and writing of the manuscript. All the authors read and approved the final manuscript.

**Financial support:** This study was funded by the Netherlands Organization for Health Research and Development (ZonMw) under grant 555 004 206.

**Potential competing interests:** P.D.S. is receiving unrestricted grants from Pentax (Japan), Norgine (UK), Motus GI (USA), MicroTech (China), and The eNose Company (Netherlands) and is in the advisory board of Motus GI (USA) and Boston Scientific (USA). All the other authors declare no competing interests.

**Data availability statement:** Both datasets and scripts used to generate the analyses in this study will be available in the DANS EASY repository.

**Study Highlights**

**WHAT IS KNOWN**

✓ Current management of Barrett's esophagus is considered ineffective in reducing mortality of esophageal adenocarcinoma.
✓ Implementation of less invasive screening tests might enable increased detection of neoplastic lesions in Barrett's esophagus.
✓ Applying a risk prediction model combining demographic, lifestyle, and clinical information may optimize preselection of a higher risk target screening population.
✓ Evidence of public perceptions of novel screening methods is sparse.

**WHAT IS NEW HERE**

✓ Intended uptake of esophageal adenocarcinoma screening is highest if a noninvasive test such as breath analysis is offered.
✓ Acceptability of a 2-step screening process with confirmatory upper endoscopy decreases if the first test is more invasive.
✓ Determining screening eligibility based on gastroesophageal reflux disease symptoms, age, or a risk prediction model would be acceptable to most individuals.
✓ Most individuals are willing to provide access to their medical file; a blood sample; and information about smoking, skin color, family history, and waist circumference as input for a risk prediction model.
✓ Using sex alone to determine screening eligibility does not receive wide support among women.

## Supplementary Material
