# Supplementary material for: The Public's Intended Uptake of Hypothetical Esophageal Adenocarcinoma Screening Scenarios: A Nationwide Survey
Source: Am J Gastroenterol. 2024 Apr 15;119(9):1802–12. doi: 10.14309/ajg.0000000000002812 (PMC11365595; doi:10.14309/ajg.0000000000002812)
Supplement: Supplementary file 1 [file acg-119-1802-s001.docx]

**Table S1.** Characteristics of participating municipalities.

| **Municipality** | **Degree of urbanisation based on address density** | **Average socio-economic status score*** | **No. of invitations sent** | **No. of included participants** | **Participation rate (%)** |
| --- | --- | --- | --- | --- | --- |
| Halderberge | Hardly urbanised | 0.054 | 749 | 241 | 32.2 |
| Vijfheerenlanden | Hardly urbanised | 0.080 | 998 | 221 | 22.1 |
| Vlaardingen | Strongly urbanised | -0.125 | 2000 | 476 | 23.8 |
| Westerwolde | Not urbanised | -0.075 | 603 | 134 | 22.2 |
| Winterswijk | Moderately urbanised | 0.004 | 2000 | 570 | 28.5 |
| Zwolle | Strongly urbanised | 0.008 | 2000 | 616 | 30.8 |

* This score is calculated by the Dutch Central Bureau for Statistics and is based on financial welfare, education attainment, and employment. The score ranges from -0.2 to 0.2, with 0 being the Dutch average.^1^
