# Supplementary figures and images for: The Public's Intended Uptake of Hypothetical Esophageal Adenocarcinoma Screening Scenarios: A Nationwide Survey
Source: Am J Gastroenterol. 2024 Apr 15;119(9):1802–12. doi: 10.14309/ajg.0000000000002812 (PMC11365595; doi:10.14309/ajg.0000000000002812)

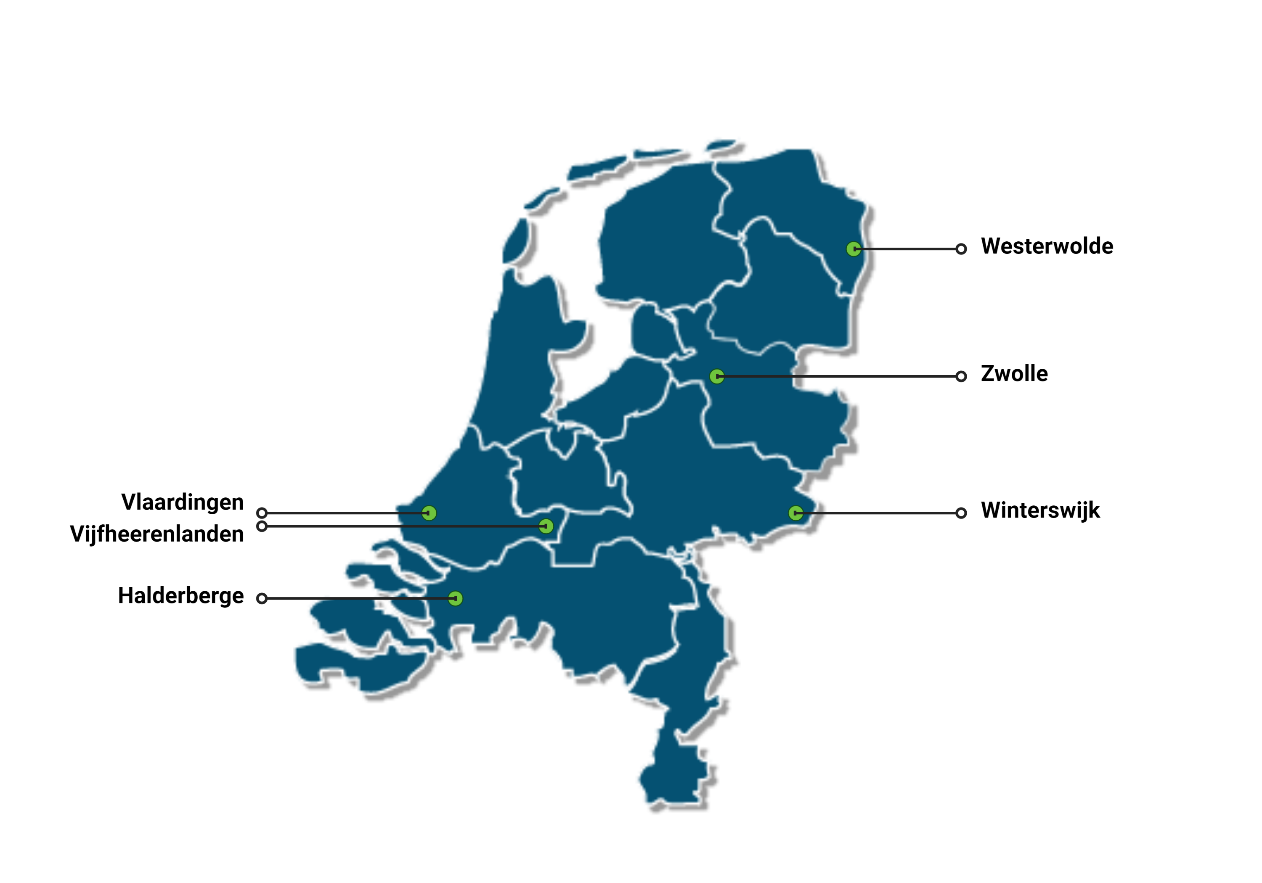


**Figure S1.** Geographic location of participating municipalities.

Supplement: Supplementary file 2 [file acg-119-1802-s002.docx]
