# Supplementary material for: The Public's Intended Uptake of Hypothetical Esophageal Adenocarcinoma Screening Scenarios: A Nationwide Survey
Source: Am J Gastroenterol. 2024 Apr 15;119(9):1802–12. doi: 10.14309/ajg.0000000000002812 (PMC11365595; doi:10.14309/ajg.0000000000002812)
Supplement: Supplementary file 3 [file acg-119-1802-s003.docx]

**Table S2.** Wording, source, and coding scheme of survey items

| **Page/**  **item no.** | **Question** | **Answer options** | **Source, validation, and coding scheme of question** | |
| --- | --- | --- | --- | --- |
| Please note that this content was delivered on the Castor EDC platform so the formatting looked slightly different to this document. The participants did not see the headings in grey boxes. | | | | |
| **Introduction page** Thank you in advance for completing this survey on early detection of esophageal cancer. Filling out the questionnaire takes 10 to 15 minutes. You can stop in between, your answers will be saved automatically. You can return to your personal survey using the URL or QR code. Some pages include images with information. Please review them carefully. You don't need to fill in any information for these. If you have any questions about completing the survey, you can contact the research team by calling (06-29061198) or sending an email to (Jasmijn.Sijben@radboudumc.nl).  You may now begin! | | | | |
| **Page 1. Informed consent**   - I have read the information sheet. I was also able to ask questions. I had enough time to decide whether or not to participate. - I know that participation is voluntary. I also know that I can decide at any time not to participate in the study or to stop participating. I don't have to explain why I want to stop. - I give permission to the researchers to collect and use my data. The researchers will only do this to answer the research questions of this study. - I am aware that certain individuals, as mentioned in the information sheet, may access all my data for the purpose of monitoring the study. I give these individuals permission to access my data for this purpose. | | | | |
| **1.1** | **I consent to the statements above.** | □ Yes  □ No | NA | |
| **Page 2. Esophageal cancer**  **Before you begin, take a look at this image to understand what esophageal cancer is and how it develops.**  *You don't need to fill out anything here*.  *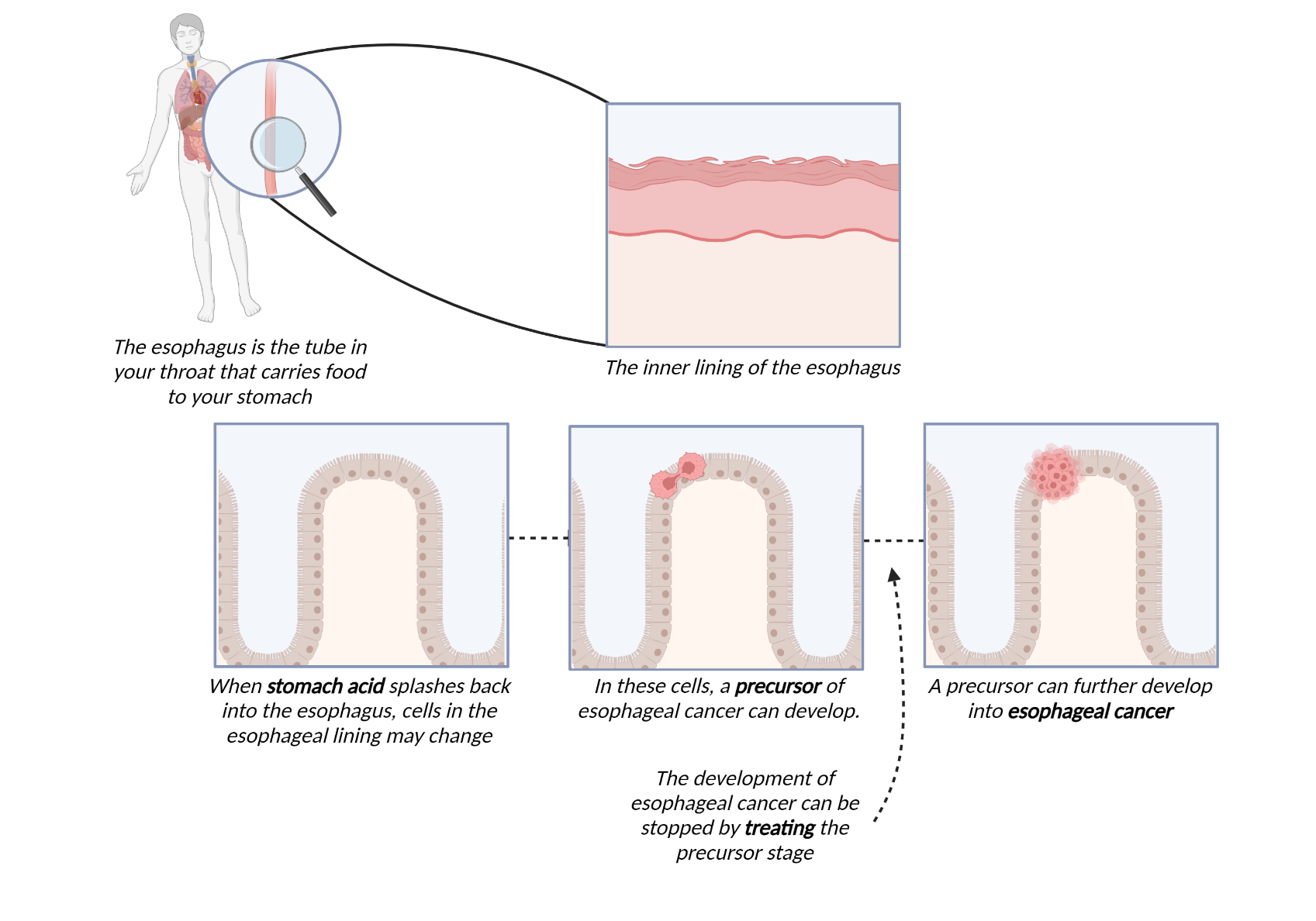* | | | | |
| **Page 3. Background and health**  In this part, we will ask you a few questions about your background and health. We won't be able to identify you based on your answers. | | | | |
| **3.1** | **What is your age?** | \|  \|  \| years \| \| --- \| --- \| --- \| | NA | |
| **3.2** | **What is your gender?** | □ Male □ Female □ Non-binary □ Other | NA | |
| **3.3** | **What is the highest level of education qualification you have obtained?** | □ Primary school (basisonderwijs)  □ Pre-vocational secondary education (VMBO)  □ Middle general secondary education (MAVO)  □ Middle vocational education (MBO)  □ Higher general secondary education or preparatory scientific education (HAVO/VWO)  □ Higher vocational education (HBO)  □ Master’s degree or doctorate  □ No education | ‘Standaard onderwijs indeling’ from ‘Statistics Netherlands (CBS)’  Education was recoded into ‘lower’ (no education/primary school/pre-vocational secondary education/middle general secondary education), ‘middle’ (middle vocational education/higher general secondary education or preparatory scientific education), and ‘higher’ (higher vocational education/master’s degree or doctorate). | |
| **3.4** | **What municipality do you live in?** | □ Halderberge □ Vijfheerenlanden □ Vlaardingen □ Westerwolde □ Winterswijk □ Zwolle □ Prefer not to say | NA | |
| **3.5** | **What is your marital status?** | □ Unmarried, single □ Unmarried, with a partner □ Married (incl civil partnership) □ Divorced □ Widowed □ Other | Marital status was recoded into ‘with a partner’ (unmarried, with a partner/married) and ‘without a partner’ (unmarries, single/divorced/ widowed/other). | |
| **3.6** | **Do you have a white skin color?** *We are asking about the color of your skin because research shows that people with a white skin color are more likely to develop esophageal cancer.* | □ Yes  □ No  □ Prefer not to say | Reviewed by ‘Pharos’ (a Dutch national expertise center that aims to reduce health disparities) | |
| 3.7 In the next part we will ask a few questions about pain or a burning feeling behind the breastbone. This area is marked in red on the image.  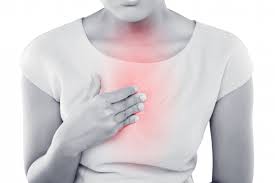  Thinking about your symptoms over the past 7 days, how often did you have the following? | | | | |
| **3.8** | **A burning feeling behind your breastbone** | □ Did not have  □ 1 day a week  □ 2 days a week  □ 3-4 days a week  □ 5-6 days a week  □ Daily | Validated Dutch translation of the Reflux Disease Questionnaire (RDQ), only the GERD domain.^2^  **Coding scheme:**   - Did not have = 0 - 1 day a week = 1 - 2 days a week = 2 - 3-4 days a week = 3 - 5-6 days a week = 4 - Daily = 5 | |
| **3.9** | **Pain behind your breastbone** |  |  |  |
| **3.10** | **An acid taste in your mouth** |  |  |  |
| **3.11** | **Unpleasant movement of material upwards from the stomach** |  |  |  |
| Shown if question 3.8 is not equal to Did not have.  Thinking about your symptoms over the past 7 days, how would you rate the following? | | | - Did not have = 0 - Very mild = 1 - Mild = 2 - Moderate = 3 - Moderately severe = 4 - Severe = 5   The RDQ mean score was calculated as the mean of the respective responses to the 8 items; RDQ mean scores thus ranged from 0 to 5. | |
| **3.8.2** | **A burning feeling behind your breastbone** | □ Very mild  □ Mild  □ Moderate  □ Moderately severe  □ Severe |  |  |
| Shown if question 3.9 is not equal to Did not have.  Thinking about your symptoms over the past 7 days, how would you rate the following? | | |  |  |
| **3.9.2** | **Pain behind your breastbone** | □ Very mild  □ Mild  □ Moderate  □ Moderately severe  □ Severe |  |  |
| Shown if question 3.10 is not equal to Did not have.  Thinking about your symptoms over the past 7 days, how would you rate the following? | | |  |  |
| **3.10.2** | **An acid taste in your mouth** | □ Very mild  □ Mild  □ Moderate  □ Moderately severe  □ Severe |  |  |
| Shown if question 3.11 is not equal to Did not have.  Thinking about your symptoms over the past 7 days, how would you rate the following? | | |  |  |
| **3.11.2** | **Unpleasant movement of material upwards from the stomach** | □ Very mild  □ Mild  □ Moderate  □ Moderately severe  □ Severe |  |  |
| We are also curious if you have experienced these symptoms before. Please fill in when you last had each symptom, if any. | | | | |
| **3.12** | **A burning feeling behind your breastbone** | □ Did not have  □ Last week  □ Last month  □ Last year  □ Longer than 1 year ago | Items 3.12 – 3.15 were based on the GERD domain of the Reflux Disease Questionnaire (RDQ).^2^ This questionnaire is not validated to measure previous GERD symptoms (more than 1 week ago). | |
| **3.13** | **Pain behind your breastbone** |  |  |  |
| **3.14** | **An acid taste in your mouth** |  |  |  |
| **3.15** | **Unpleasant movement of material upwards from the stomach** |  |  |  |
| **3.16** | **Have you ever had an endoscopy of your esophagus and stomach?** | □ Yes □ No □ Don’t know | From discrete choice experiment about intended uptake of EAC screening, Peters et al., 2020^3^ | |
| **3.17** | **Have you ever had any form of cancer?** | □ Yes  □ No | Not validated | |
| **3.17.1** | Shown if question 3.17 is equal to ‘Yes’:  **Have you ever had esophageal cancer?** | □ Yes  □ No | Not validated | |
| **3.18** | **Have your biological parents or any of your siblings ever had esophageal cancer?** | □ Yes □ No □ Don’t know | Not validated | |
| **3.19** | **Do you know anyone outside your biological family who has or had esophageal cancer?** | □ Yes  □ No | Not validated | |
| **Page 4. Beliefs about cancer and early detection**  In this part, we will ask some questions regarding your beliefs about cancer and population screening for cancer. | | | | |
| **4.1** | **How often have you been worried about your chances of getting cancer?** *This question concerns all types of cancer* | □ Almost never □ Sometimes □ Often □ Almost all the time | Dutch translation of Lerman’s Cancer Worry scale – severity domain.^4,5^ | |
| **4.2** | **Compared to the average person your age, would you say that you are more likely to get esophageal cancer, less likely, or about as likely?** | □ More likely □ About as likely □ Less likely | Adapted from the Health Information National Trends Surveys (HINTS) 2008. HINTS are population-based surveys of the civilian non-institutionalized population of the United States. | |
| **4.3** | Shown if eligible for bowel cancer screening according to age and sex:  **Have you had a bowel cancer screening test in the past 2 years?** | □ Yes □ No □ I did not receive an invitation | Adapted from the internationally validated Awareness and Beliefs about Cancer (ABC) measure: cancer screening module.^6^ The items about cancer screening behavior were made specific for centralized screening programs in the Netherlands. | |
| **4.4** | Shown if eligible for breast cancer screening according to age and sex:  **Have you had a breast cancer screening test, mammogram, in the past 3 years?** |  |  |  |
| **4.5** | Shown if eligible for cervical cancer screening according to age and sex:  **Have you had a cervical cancer screening test in the past 10 years?** |  |  |  |
| Below are 3 statements about population screening for cancer. Please indicate how much you agree or disagree with each item. | | | | |
| **4.6** | **I would be so worried about what might be found at cancer screening that I would prefer not to have it.** | □ Strongly disagree □ Tend to disagree □ Tend to agree □ Strongly agree | Adapted from the internationally validated Awareness and Beliefs about Cancer (ABC) measure: cancer screening module.^6^ | |
| **4.7** | **Cancer screening is only necessary if I have symptoms.** |  |  |  |
| **4.8** | **Cancer screening could reduce my chance of dying from breast cancer.** |  |  |  |
| **Page 5. Gastroscopy**  A gastroscopy is a camera test used to investigate if someone has cancer in their esophagus. The images below show how this is done.  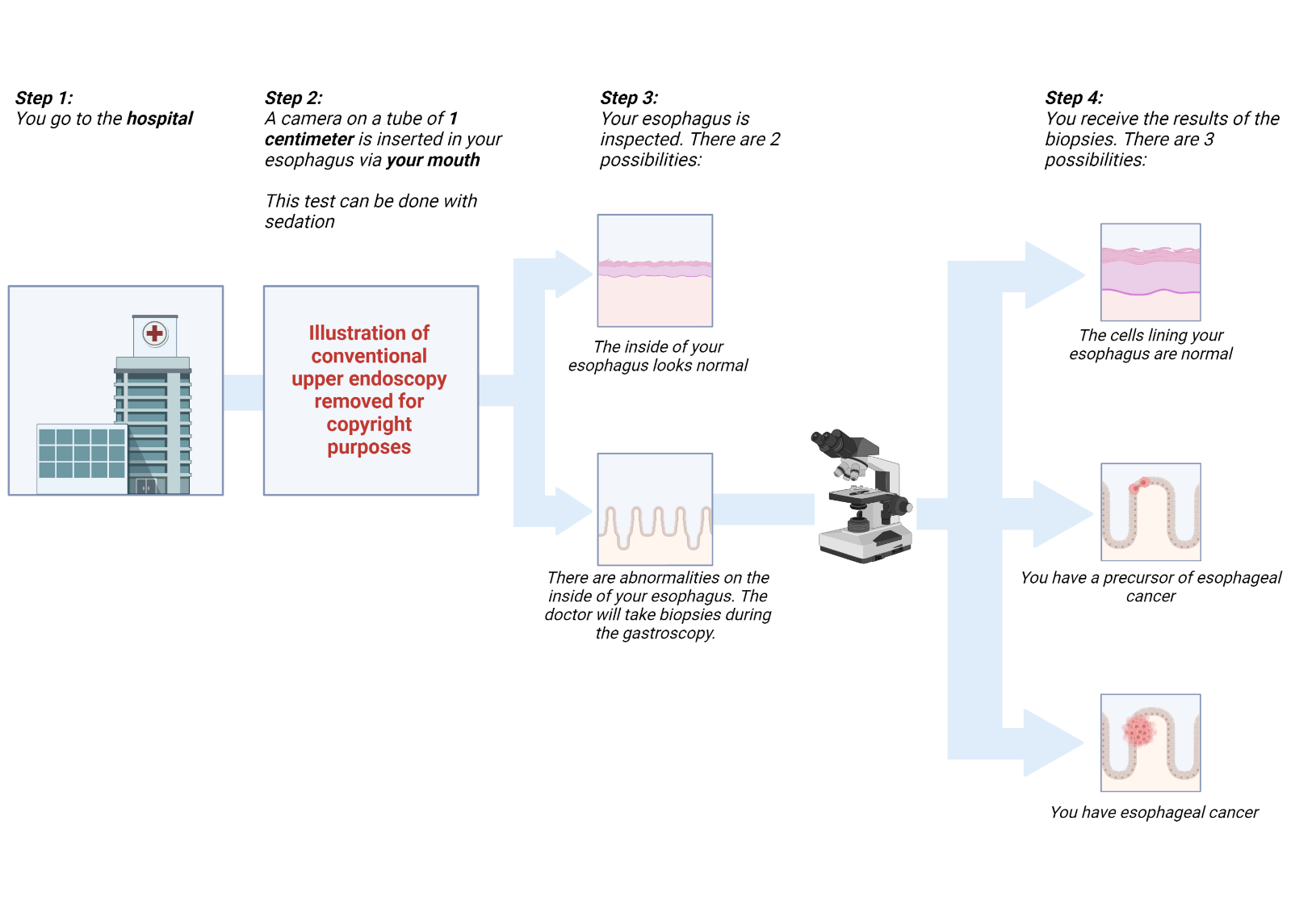  The questions with a slider below can be answered on a scale from 1 to 7. | | | | |
| **5.1** | **Do you expect to experience physical discomfort during the gastroscopy?** | Slider ranging from 1 (no discomfort at all) to 7 (extreme discomfort) | Likert-type scales have previously been validated for measuring pain severity;^7^ but not for gagging and general physical discomfort. | |
| **5.2** | **Do you expect to experience gagging during the gastroscopy?** | Slider ranging from 1 (no gagging at all) to 7 (extreme gagging) |  |  |
| **5.3** | **If you were invited to have a gastroscopy as part of a screening program for esophageal cancer, would you participate?** | □ Yes, absolutely □ Yes, I think so □ No, I don’t think so □ No, absolutely not | Adapted from the bowel cancer screening literature, Hawranek et al., 2022.^8^ | |
| **Page 6. Nasal endoscopy**  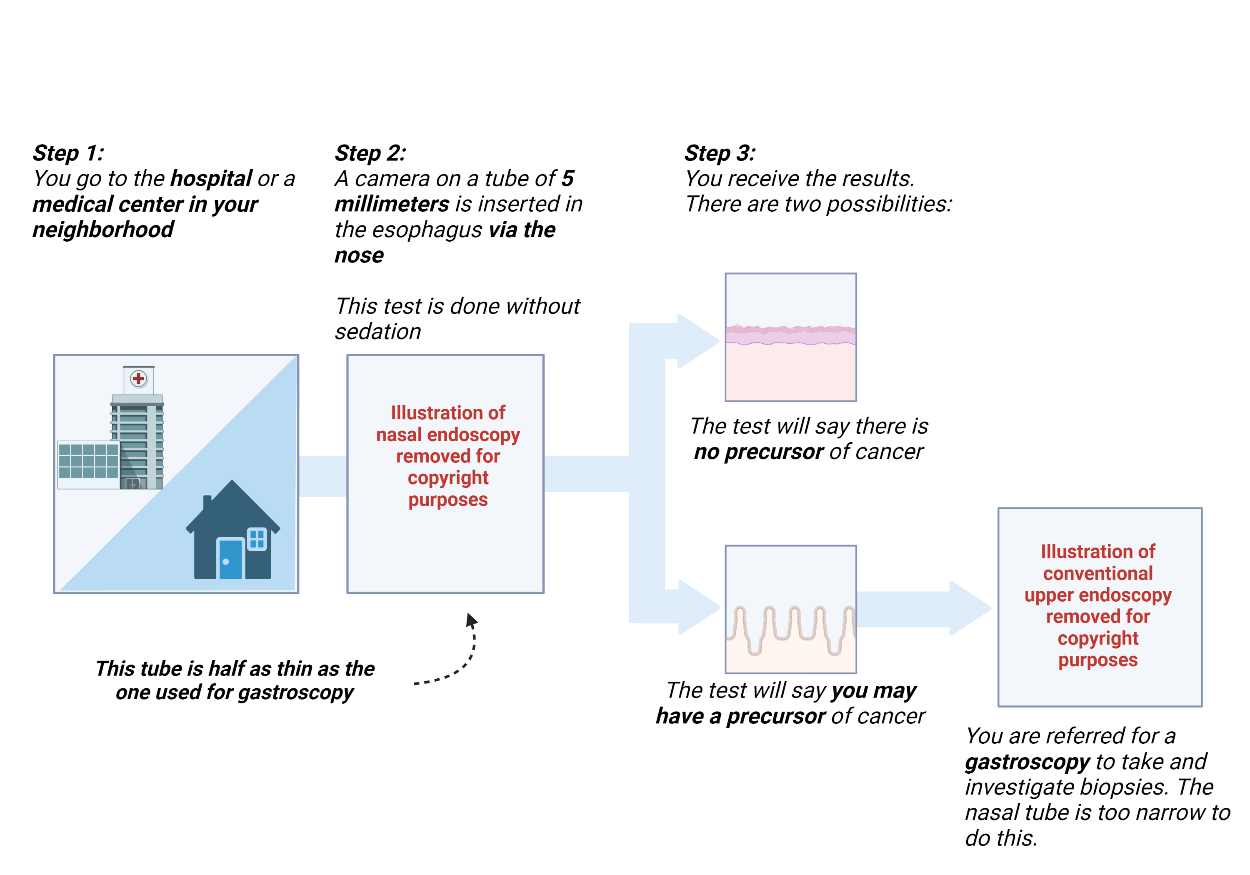Researchers are investigating other ways to detect esophageal cancer, for example through a **nasal endoscopy**. You can see how this is done on the image below. | | | | This version of page 6 was shown to participants randomized in the transnasal endoscopy scenario, but not to participants randomized in the ingestible cell-collection device or breath test scenario. |
| **6.1** | **Do you expect to experience physical discomfort during the nasal endoscopy?** | Slider ranging from 1 (no discomfort at all) to 7 (extreme discomfort) | See item 5.1 and 5.2 | |
| **6.2** | **Do you expect to experience gagging during the nasal endoscopy?** | Slider ranging from 1 (no gagging at all) to 7 (extreme gagging) |  |  |
| **Sometimes the result of nasal endoscopy is not reliable:**  **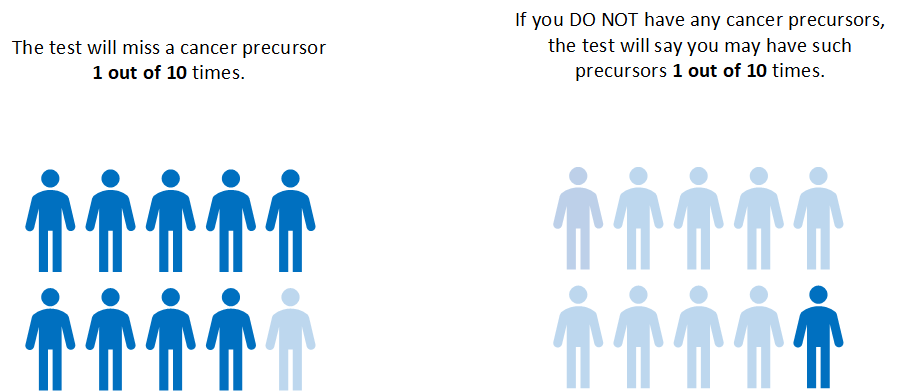** | | | | |
| **6.3** | **Do you think the reliability of nasal endoscopy is acceptable?** *Please read the information on the image above* | Slider ranging from 1 (not acceptable at all) to 7 (extremely acceptable) | Item based on focus group results (not validated) and information about test performance based on meta-analysis by Huibertse et al.^9^ | |
| **6.4** | **If you were invited to have a nasal endoscopy as part of a screening program for esophageal cancer, would you participate?** | □ Yes, absolutely □ Yes, I think so □ No, I don’t think so □ No, absolutely not | See item 5.3 | |
| **6.8.1** | Shown if question 6.4 is not equal to ‘No, absolutely not’. **Please imagine that you participated and the nasal endoscopy said you may have a precursor of esophageal cancer. Further testing by means of a gastroscopy is needed to provide a final result. This is because the tube used for the nasal endoscopy is too narrow to take reliable samples. Do you think it's acceptable to have 2 tests (nasal endoscopy and gastroscopy) to get a final result?** | □ Yes □ No □ Don’t know | Based on focus group results (not validated) | |
| **Page 6. Pill on a string test**  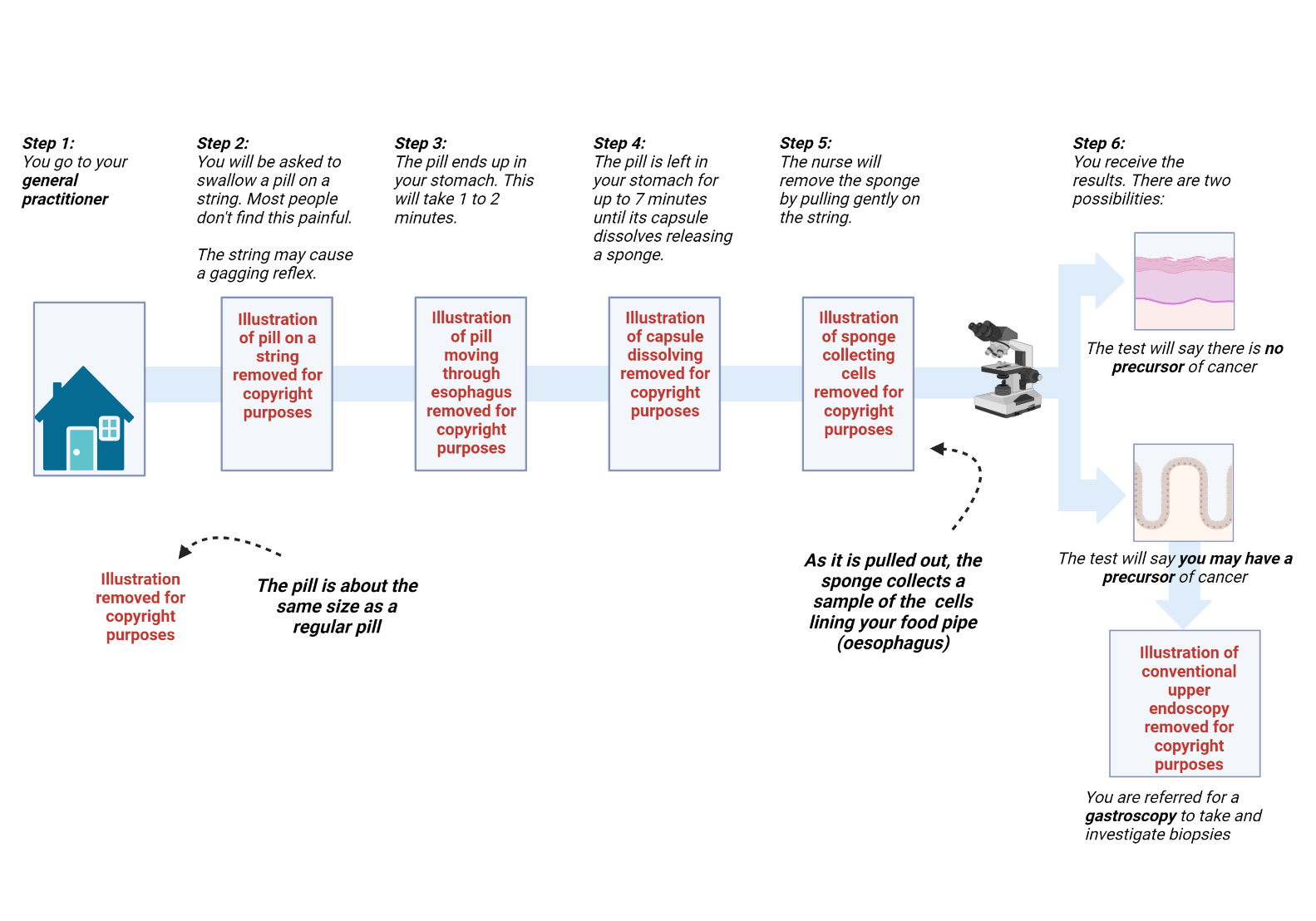Researchers are investigating other ways to detect esophageal cancer, for example through a  **pill on a string test**. You can see how this is done on the image below. | | | This version of page 6 was shown to participants randomized in the ingestible cell-collection device scenario, but not to participants randomized in the transnasal endoscopy or breath test scenario. | |
| **6.1** | **Do you expect to experience physical discomfort during the pill on a string test?** | Slider ranging from 1 (no discomfort at all) to 7 (extreme discomfort) | See item 5.1 and 5.2 | |
| **6.2** | **Do you expect to experience gagging during the pill on a string test?** | Slider ranging from 1 (no gagging at all) to 7 (extreme gagging) |  |  |
| **Sometimes the result of the pill on a string test is not reliable:**  **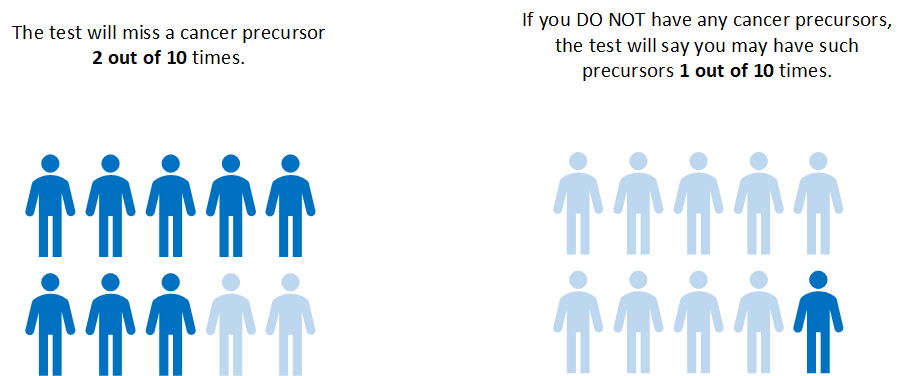** | | | | |
| **6.3** | **Do you think the reliability of the pill on a string test is acceptable?** *Please read the information on the image above* | Slider ranging from 1 (not acceptable at all) to 7 (extremely acceptable) | Item based on focus group results (not validated) and information about test performance based on study by Ross-Ines et al.^10^ | |
| **6.4** | **If you were invited to have the pill on a string test as part of a screening program for esophageal cancer, would you participate?** | □ Yes, absolutely □ Yes, I think so □ No, I don’t think so □ No, absolutely not | See item 5.3 | |
| **6.4.1** | Shown if question 6.4 is not equal to ‘No, absolutely not’.  **Please imagine that you participated and the pill on a string test said you may have a precursor of esophageal cancer. Further testing by means of a gastroscopy is needed to provide a final result. This is because the tube used for the nasal endoscopy is too narrow to take reliable samples. Do you think it's acceptable to have 2 tests (pill on a string test and gastroscopy) to get a final result?** | □ Yes □ No □ Do not know | Based on focus group results (not validated) | |
| **Page 6. Breath test**  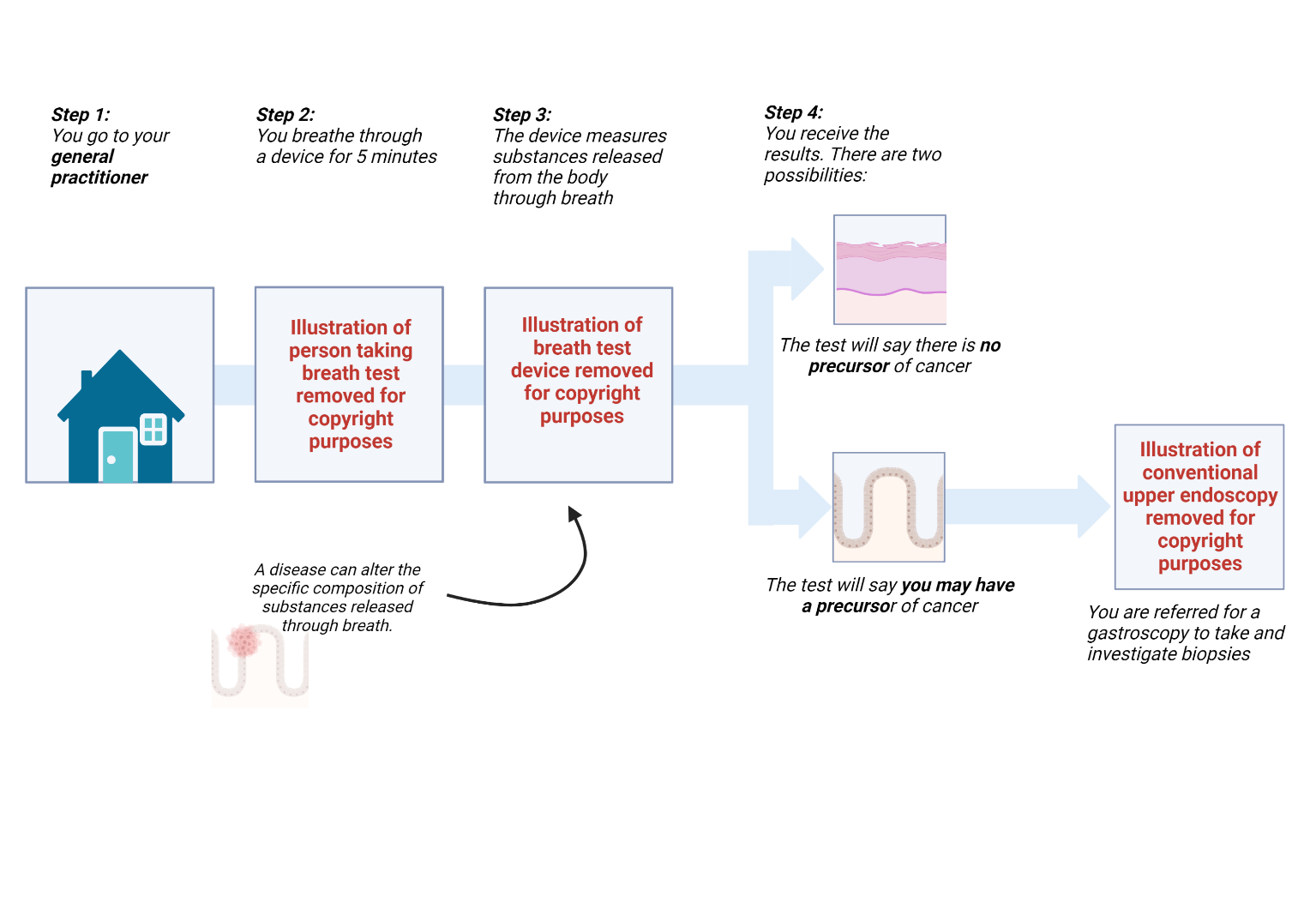Researchers are investigating other ways to detect esophageal cancer, for example through a **breath test**. You can see how this is done on the image below | | | This version of page 6 was shown to participants randomized in the breath test scenario, but not to participants randomized in the transnasal endoscopy or ingestible cell-collection device scenario. | |
| **6.1** | **Do you expect to experience physical discomfort during the breath test?** | Slider ranging from 1 (no discomfort at all) to 7 (extreme discomfort) | See item 5.1 and 5.2 | |
| **6.2** | **Do you expect to experience gagging during the breath test?** | Slider ranging from 1 (no gagging at all) to 7 (extreme gagging) |  |  |
| **Sometimes the result of the breath test is not reliable:**  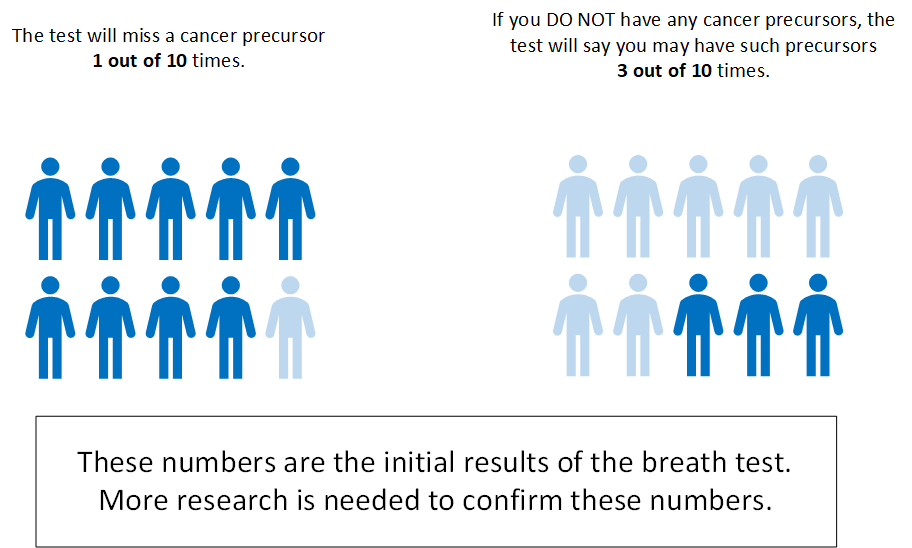  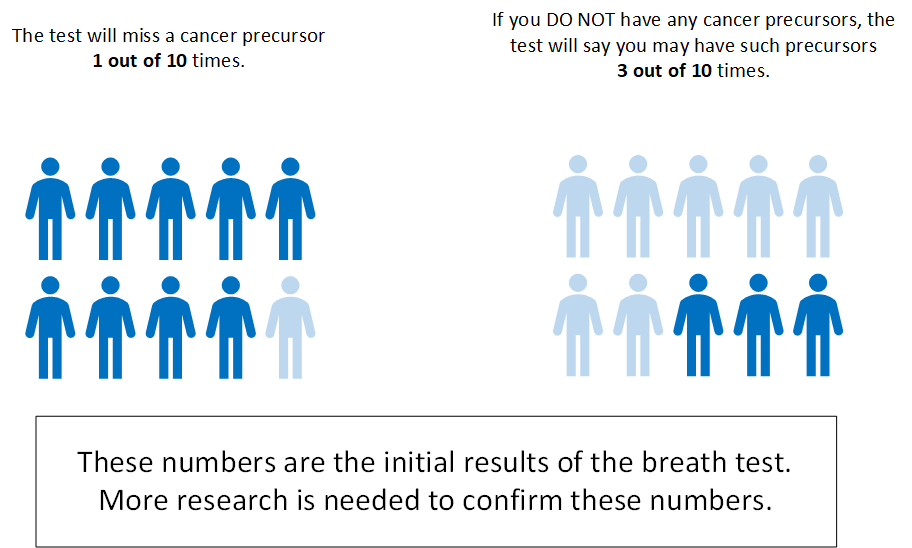 | | | | |
| **6.3** | **Do you think the reliability of nasal endoscopy is acceptable?** *Please read the information on the image above* | Slider ranging from 1 (not acceptable at all) to 7 (extremely acceptable) | Item based on focus group results (not validated) and information about test performance based on study by Peters et al.^11^ | |
| **6.4** | **If you were invited to have a breath test as part of a screening program for esophageal cancer, would you participate?** | □ Yes, absolutely □ Yes, I think so □ No, I don’t think so □ No, absolutely not | See item 5.3 | |
| **6.4.1** | Shown if question 6.4 is not equal to ‘No, absolutely not’.  **Please imagine that you participated and the breath test said you may have a precursor of esophageal cancer. Further testing by means of a gastroscopy is needed to provide a final result. This is because the tube used for the nasal endoscopy is too narrow to take reliable samples. Do you think it's acceptable to have 2 tests (breath test and gastroscopy) to get a final result?** | □ Yes □ No □ Don’t know | Based on focus group results (not validated) | |
| **Page 7. Target screening population**  Esophageal cancer affects about 3000 adults each year in the Netherlands. If we would screen all 14 million adults, most of them would not benefit from the screening program. One way of increasing the number of people who benefit is to only offer screening to people who we think are more likely to develop esophageal cancer. Please consider the following situations and answer the questions honestly, we would like to know YOUR opinion.  **Situation 1.** Let’s say you sometimes experience heartburn. You receive a letter inviting you to esophageal cancer screening. The letter states that ‘only people at an age of 45 years or older who experience heartburn are invited because esophageal cancer is more common in this group’. The letter then explains that you were invited based on information registered in your medical file at the GP’s office. | | | | |
| **7.1** | **How acceptable does it seem to you that health care professionals use age to decide when you should start esophageal cancer screening?** | Slider ranging from 1 (not acceptable at all) to 7 (extremely acceptable) | The item format for questions 7.1 – 7.10 was taken from Usher et al., 2021,^12^ and made specific to potential risk stratification approaches for esophageal cancer screening^13-16^ (not validated) | |
| **7.2** | **How acceptable does it seem to you that only people who experience heartburn would be offered screening?** |  |  |  |
| **7.3** | **Would you be willing to provide access to your medical file for this purpose?** | Slider ranging from 1 (not willing at all) to 7 (extremely willing) |  |  |
| Shown if question 3.2 is not equal to Female.  **Situation 2.** Now imagine that you receive a letter inviting you to esophageal cancer screening and it states that you are being invited because ‘you are a man and men are 2-3 times more likely to develop esophageal cancer than women. | | | | |
| **7.4** | **How acceptable does it seem to you that men would be offered screening and women would not?** | Slider ranging from 1 (not acceptable at all) to 7 (extremely acceptable) |  | |
| Shown if question 3.2 is Female.  **Situation 2.** Now imagine that you did not receive an invitation but a male friend or relative who is the same age as you receives a letter inviting them to esophageal cancer screening. In that letter it states that it is because ‘men are 2-3 times more likely to develop esophageal cancer than women.’ You will not receive an invitation for screening because you are a woman. | | | | |
| **7.4** | **How acceptable does it seem to you that men would be offered screening and women would not?** | Slider ranging from 1 (not acceptable at all) to 7 (extremely acceptable) |  | |
| **Situation 3.** Your personal risk of esophageal cancer can be estimated by a calculator.  **Y**ou can see how this works on the image below**.**  **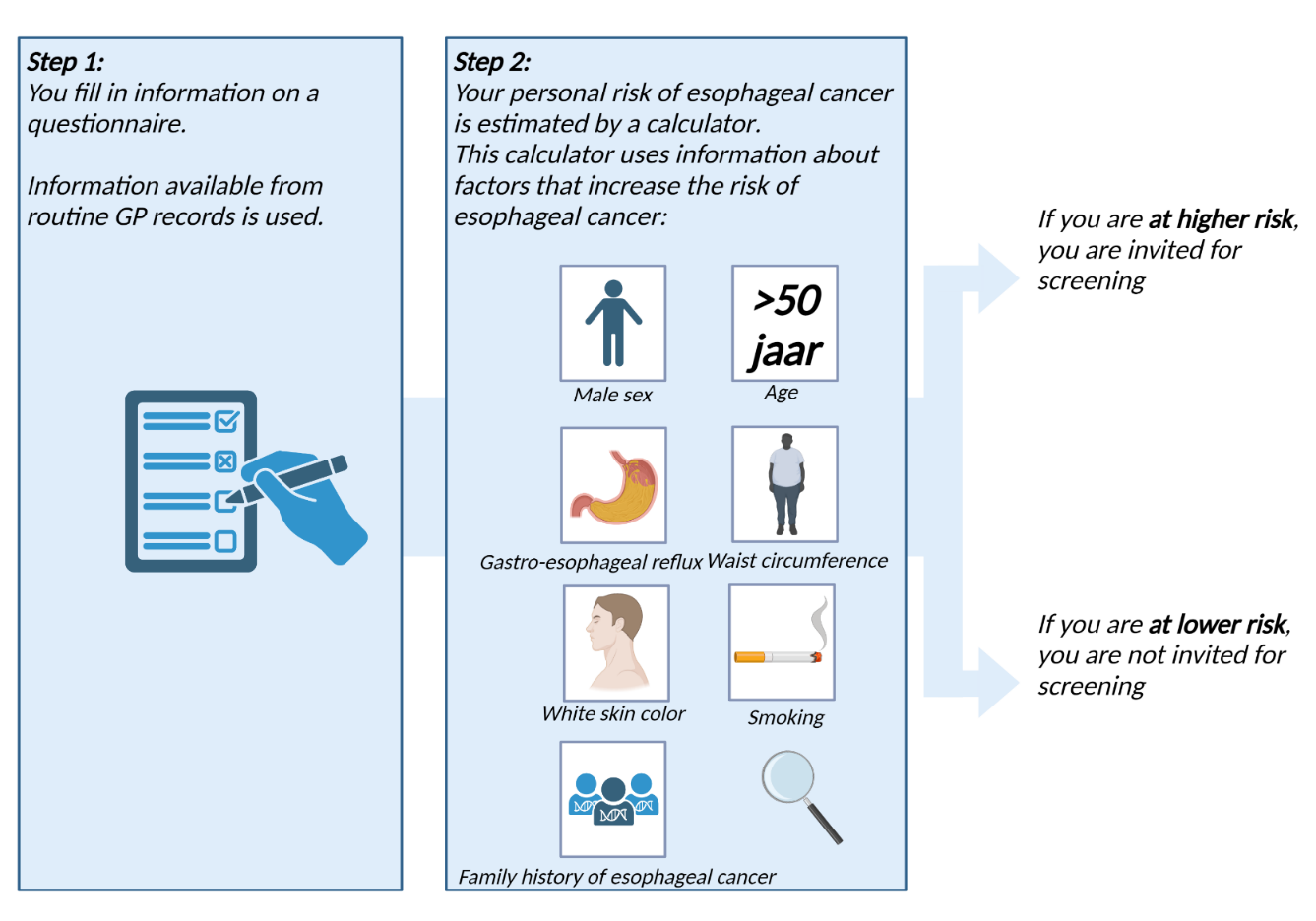** | | | | |
| **7.5** | **How acceptable does it seem to you that this calculator is used to determine if you are eligible for esophageal cancer screening?** | Slider ranging from 1 (not acceptable at all) to 7 (extremely acceptable) |  | |
| **7.6** | **Are you willing to provide information about your waist circumference to determine if you are eligible for esophageal cancer screening?** | Slider ranging from 1 (not willing at all) to 7 (extremely willing) |  | |
| **7.7** | **Are you willing to provide information about your skin color to determine if you are eligible for esophageal cancer screening?** |  |  |  |
| **7.8** | **Are you willing to provide information about your lifestyle (smoking) to determine if you are eligible for esophageal cancer screening?** |  |  |  |
| **7.9** | **Are you willing to provide information about your family history to determine if you are eligible for esophageal cancer screening?** |  |  |  |
| **Situation 4.** Now imagine the results of a blood test are added to the calculator that estimates your personal risk of esophageal cancer. | | | | |
| **7.10** | **Would you be willing to provide a sample of blood to determine if you are eligible for esophageal cancer screening?** | Slider ranging from 1 (not willing at all) to 7 (extremely willing) |  | |
| **Page 8. Organization**  If you were invited for esophageal cancer screening, how would each of the following influence your decision whether to take up the invitation? | | | | |
| **8.1** | **If it was recommended by your GP?** | □ Much less likely to attend □ Slightly less likely to attend  □ No influence  □ Slightly more likely to attend  □ Much more likely to attend | Barriers and facilitators were based on literature review and focus group results, item format was taken from Harvey-Kelly et al., 2020^17^ (not validated). | |
| **8.2** | **If you could do the test at home?** |  |  |  |
| **8.3** | **If you could do the test at the GP?** |  |  |  |
| **8.4** | **If you had to go to the hospital for the test?** |  |  |  |
| **8.5** | **If you received a reminder to make an appointment?** |  |  |  |
| **8.6** | **If you had to pay for the test?** |  |  |  |
| **8.7** | **If you were busy/did not have a lot of time?** |  |  |  |
| 8**.8** | **What do you think is the best way to be invited for esophageal cancer screening?** | □ During a conversation with my GP  □ A letter from my GP  □ A letter from a public health organization (Bevolkingsonderzoek Nederland) | Based on focus group results (not validated) | |
| **8.9** | **Would you prefer to consult a healthcare professional before making a decision about participating in esophageal cancer screening?** | □ Not needed  □ Yes, with my GP □ Yes, with someone from a public health organization □ Yes, does not matter with whom  □ Do not know | Based on focus group results (not validated) | |
| Please read the following information about risk factors and warning signs of esophageal cancer.  **Risk factors** are things that (slightly) increase the chance of developing esophageal cancer, such as: older age, male gender, experiencing heartburn, large waist circumference, smoking, white skin, and a family history of esophageal cancer.  **Warning signs** are symptoms that could suggest the presence of esophageal cancer, for example: difficulty swallowing food or unexplained weight loss. | | | | |
| **8.10** | **Do you think that information about the risk factors and warning signs of esophageal cancer should be disseminated?** | □ No □ Yes, but only about risk factors  □ Yes, but only about alarm signals □ Yes, about risk factors and alarm signals  □ I do not have a clear opinion on this | Based on focus group results (not validated) | |
| **8.11** | **How much do you agree with the following: ‘Spending money on esophageal cancer screening is a waste of the healthcare budget.** | □ Strongly disagree □ Disagree □ Agree □ Strongly agree | Item was adapted from the Cancer Stigma Scale.^18^ | |
| *Thank you for completing the survey. Your time and responses are very much appreciated.* | | | | |

**References**

1. Sociaal-economische status per postcode, 2019. (Statistics Netherlands, <https://www.cbs.nl/nl-nl/maatwerk/2022/26/sociaal-economische-status-per-postcode-2019> (accessed 15 May 2023)).

2. Aanen, M.C., Numans, M.E., Weusten, B.L. & Smout, A.J. Diagnostic value of the Reflux Disease Questionnaire in general practice. *Digestion* **74**, 162-168 (2006).

3. Peters, Y. & Siersema, P.D. Public Preferences and Predicted Uptake for Esophageal Cancer Screening Strategies: A Labeled Discrete Choice Experiment. *Clin Transl Gastroenterol* **11**, e00260 (2020).

4. Lerman, C.*, et al.* Psychological side effects of breast cancer screening. *Health Psychol* **10**, 259-267 (1991).

5. Douma, K.F.*, et al.* Psychological distress and use of psychosocial support in familial adenomatous polyposis. *Psychooncology* **19**, 289-298 (2010).

6. Simon, A.E.*, et al.* An international measure of awareness and beliefs about cancer: development and testing of the ABC. *BMJ Open* **2**(2012).

7. Todd, K.H., Funk, K.G., Funk, J.P. & Bonacci, R. Clinical significance of reported changes in pain severity. *Ann Emerg Med* **27**, 485-489 (1996).

8. Hawranek, C.*, et al.* Cancer Worry Distribution and Willingness to Undergo Colonoscopy at Three Levels of Hypothetical Cancer Risk-A Population-Based Survey in Sweden. *Cancers (Basel)* **14**(2022).

9. Huibertse, L.J., Peters, Y., Westendorp, D. & Siersema, P.D. Unsedated transnasal endoscopy for the detection of Barrett's esophagus: systematic review and meta-analysis. *Dis Esophagus* (2022).

10. Ross-Innes, C.S.*, et al.* Evaluation of a minimally invasive cell sampling device coupled with assessment of trefoil factor 3 expression for diagnosing Barrett's esophagus: a multi-center case-control study. *PLoS Med* **12**, e1001780 (2015).

11. Peters, Y.*, et al.* Detection of Barrett's oesophagus through exhaled breath using an electronic nose device. *Gut* **69**, 1169-1172 (2020).

12. Usher-Smith, J.A.*, et al.* Acceptability and potential impact on uptake of using different risk stratification approaches to determine eligibility for screening: A population-based survey. *Health Expect* **24**, 341-351 (2021).

13. Shaheen, N.J.*, et al.* Diagnosis and Management of Barrett's Esophagus: An Updated ACG Guideline. *Am J Gastroenterol* **117**, 559-587 (2022).

14. Fitzgerald, R.C.*, et al.* Cytosponge-trefoil factor 3 versus usual care to identify Barrett's oesophagus in a primary care setting: a multicentre, pragmatic, randomised controlled trial. *Lancet* **396**, 333-344 (2020).

15. Rubenstein, J.H.*, et al.* Validation of Tools for Predicting Incident Adenocarcinoma of the Esophagus or Esophagogastric Junction. *Am J Gastroenterol* **116**, 949-957 (2021).

16. Rubenstein, J.H.*, et al.* Endoscopic Screening Program for Control of Esophageal Adenocarcinoma in Varied Populations: A Comparative Cost-Effectiveness Analysis. *Gastroenterology* **163**, 163-173 (2022).

17. Harvey-Kelly, L.L.W.*, et al.* Public attitudes towards screening for kidney cancer: an online survey. *BMC Urol* **20**, 170 (2020).

18. Marlow, L.A. & Wardle, J. Development of a scale to assess cancer stigma in the non-patient population. *BMC Cancer* **14**, 285 (2014).
