# Supplementary material for: The Public's Intended Uptake of Hypothetical Esophageal Adenocarcinoma Screening Scenarios: A Nationwide Survey
Source: Am J Gastroenterol. 2024 Apr 15;119(9):1802–12. doi: 10.14309/ajg.0000000000002812 (PMC11365595; doi:10.14309/ajg.0000000000002812)
Supplement: Supplementary file 4 [file acg-119-1802-s004.docx]

**Sensitivity analyses**

**Methods**

The Dutch Statistics Bureau provides publicly available distributions of the general population’s age, gender, education level, and civil status.^1^ The proportion of individuals with a personal history of cancer within the Netherlands is ascertainable through the Netherlands Cancer Registration.^2^ Additionally, Key Performance Indicators from the Dutch cancer screening program provide statistics of the proportion of women and men invited to participate in breast, cervical, or bowel cancer screenings.^3^ Despite knowing proportions for the entire population across these variables, the absence of individual-level data precludes the generation of a cross-table incorporating the desired ratios. Instead, we systematically excluded participants from overrepresented groups in a stepwise manner to arrive at a subsample with characteristics (i.e., age, gender, education, civil status and personal history of cancer) matching the distributions in the general population (subsample 1). We subsequently conducted a sensitivity analysis with this subsample.

The 'Participated in other cancer screening' variable already contains a selected sample of the entire population, as it only includes individuals eligible for any screening program in the denominator. Given that cancer screening eligibility is based on gender and age, selecting a subsample of our participants that matches the general population in terms of age, gender, and prior participation in cancer screening is impossible. Therefore, a second sensitivity analysis was executed with a subsample matching the proportion of cancer screening participation in the general population (**subsample 2**).

*Selection subsample 1*
We initiated this process with the variable that would most likely influence the proportions in other non-representative variables (age group).
The age distributions in the original study sample and general population are as follows:

**Study variable:** age groups

| **Original study sample (n=2258)** | | | | **Dutch population aged 45-74** | | |
| --- | --- | --- | --- | --- | --- | --- |
| 45-54y n (%) | 55-64y n (%) | 65-74y n (%) | Missing n(%) | 45-54y (%) | 55-64 y (%) | 65-74 y (%) |
| 627 (27.8) | 832 (36.8) | 797 (35.3) | 2 (0.1) | 35.1 | 35.9 | 29.0 |

We calculated the ratio between the distribution of age groups in the general Dutch population:

35.1 : 35.9 : 29.0
1 : 1.03 : 0.83

We use this ratio to calculate how many individuals should be retained in each age group:

1 : 1.03 : 0.83
627 : 641 : 518

832 – 641 = 191 need to be removed from the 55-64 age group in the study sample AND
797 – 518 = 279 need to be removed from the 65-74 group in study sample to arrive at a proportion representative for the Dutch population:

| **Study subsample 1.1 (n=1788)** | | | | **Dutch population aged 45-74** | | |
| --- | --- | --- | --- | --- | --- | --- |
| 45-54y n (%) | 55-64y n (%) | 65-74y n (%) | Missing n(%) | 45-54y (%) | 55-64 y (%) | 65-74 y (%) |
| 627 (35.1) | 641 (35.9) | 518 (28.9) | 2 (0.1) | 35.1 | 35.9 | 29.0 |

To randomly remove 191 participants from the 55-64 age group, we first assigned a random number to each participant in this age group using the function ‘RandomNumbers=RV.UNIFORM’ in SPSS. Subsequently, we ordered and ranked participants based on the random number and excluded the first 191 participants. We repeated this step to remove participants from the 65-74 age group.

We repeated this process to, respectively, exclude individuals from middle and higher education groups, with prior cancer history and who were married, to arrive at subsample 1.

We used the same method to calculate how many individuals with previous participation in cancer screening should be removed from the original sample to select subsample 2.

**Supplemental Table S3.** Baseline characteristics subsample 1.

|  | **Overall** | | | **Transnasal endoscopy scenario** | | **Ingestible cell-collection device scenario** | | **Breath test scenario** | |
| --- | --- | --- | --- | --- | --- | --- | --- | --- | --- |
|  | **Original sample (N=2258)** | **Subsample 1 (N=1104)** | **Dutch population** | **Original sample (N=539)** | **Subsample 1 (N=265)** | **Original sample (N=547)** | **Subsample 1 (N=287)** | **Original sample (N=1172)** | **Subsample 1 (N=552)** |
| 45 – 54, n (%) | 627 (27.8) | 397 (36.0) | 35.1 | 132 (24.5) | 88 (33.2) | 163 (29.8) | 113 (39.4) | 332 (28.3) | 196 (35.5) |
| 55 – 64, n (%) | 832 (36.8) | 383 (34.7) | 35.9 | 207 (38.4) | 97 (36.6) | 204 (37.3) | 98 (34.1) | 421 (35.9) | 188 (34.1) |
| 65 – 74, n (%) | 797 (35.3) | 322 (29.2) | 29.0 | 199 (36.9) | 79 (29.8) | 179 (32.7) | 75 (26.1) | 419 (35.8) | 168 (30.4) |
| Female, n (%) | 1127 (49.9) | 560 (50.7) | 50.3 | 265 (49.2) | 142 (53.6) | 267 (48.8) | 136 (47.4) | 595 (50.8) | 282 (51.1) |
| Education level, n (%) |  |  |  |  |  |  |  |  |  |
| Lower | 535 (23.7) | 295 (26.7) | 27.8 | 129 (23.9) | 66 (24.9) | 126 (23.0) | 71 (24.7) | 280 (23.9) | 158 (28.6) |
| Middle | 857 (38.0) | 434 (39.3) | 37.6 | 194 (36.0) | 102 (38.5) | 217 (39.7) | 113 (39.4) | 446 (38.1) | 219 (39.7) |
| Higher | 866 (38.4) | 375 (34.0) | 33.6 | 216 (40.1) | 97 (36.6) | 204 (37.3) | 103 (35.9) | 446 (38.1) | 175 (31.7) |
| Civil status, n married (%) | 1598 (70.8) | 686 (62.1) | 62.3 | 368 (68.3) | 150 (56.6) | 384 (70.2) | 178 (62.0) | 846 (72.2) | 358 (64.9) |
| Personal history of cancer, n yes (%) | 284 (12.6) | 72 (6.5) | 7.2 | 70 (13.0) | 19 (7.2) | 69 (12.6) | 24 (8.4) | 145 (12.4) | 29 (5.3) |
| Participated in other cancer screening, n yes (%) | 1726 (88.2)^a^ | 799 (87.8)^b^ | 67.6 | 427 (90.1) | 201 (90.1) | 398 (82.0) | 198 (88.0) | 901 (89.2) | 400 (88.3) |

^a^ Among individuals eligible for any program (n= 1958).
^b^ Among individuals eligible for any program (n= 910).

**Supplemental Table S4.** Anticipated discomfort, acceptability test performance, and intended uptake of screening scenarios in subsample 1.

|  | **Upper endoscopy scenario** | | **Transnasal endoscopy scenario** | | **Ingestible cell-collection device scenario** | | **Breath test scenario** | |
| --- | --- | --- | --- | --- | --- | --- | --- | --- |
|  | **Original sample (N = 2258)** | **Subsample 1 (N=1104)** | **Original sample (N = 539)** | **Subsample 1 (N=265)** | **Original sample (N = 547)** | **Subsample 1 (N=287)** | **Original sample  (N = 1172)** | **Subsample 1 (N=552)** |
| Anticipated discomfort, m (SD) | 5.1 (1.6) | 5.1 (1.6) | 4.7 (1.5) | 4.7 (1.5) | 4.0 (1.6) | 4.0 (1.7) | 1.5 (0.9) | 1.5 (0.9) |
| Anticipated gagging, m (SD) | 5.1 (1.6) | 5.1 (1.6) | 4.1 (1.7) | 4.0 (1.7) | 4.2 (1.7) | 4.2 (1.8) | 1.5 (1.0) | 1.5 (0.9) |
| Acceptability test performance, m (SD) | n/a | n/a | 4.0 (1.5) | 3.9 (1.5) | 4.2 (1.4) | 4.1 (1.4) | 4.3 (1.5) | 4.3 (1.4) |
| Intended participation, n(%) |  |  |  |  |  |  |  |  |
| Yes, absolutely | 785 (34.8) | 379 (34.3) | 135 (25.0) | 69 (26.0) | 165 (30.2) | 76 (26.5) | 756 (64.5) | 355 (64.3) |
| Yes, I think so | 973 (43.1) | 493 (44.7) | 232 (43.0) | 122 (46.0) | 244 (44.6) | 144 (50.2) | 361 (30.8) | 176 (31.9) |
| No, I don’t think so | 411 (18.2) | 189 (17.1) | 131 (24.3) | 55 (20.8) | 112 (20.5) | 54 (18.8) | 36 (3.1) | 12 (2.2) |
| No, absolutely not | 60 (2.7) | 27 (2.4) | 27 (5.0) | 10 (3.8) | 8 (1.5) | 5 (1.7) | 5 (0.4) | 1 (0.2) |
| Missing | 29 (1.32 | 16 (1.4) | 14 (2.7) | 9 (3.4) | 18 (3.2) | 8 (2.8) | 14 (1.2) | 8 (1.4) |

**Supplemental Table S5.** Acceptability eligibility criteria subsample 1.

|  | **Original sample (N = 2258)** | **Subsample 1 (N=1104)** |
| --- | --- | --- |
| GERD, n acceptable (%) | 1655 (73.3) | 804 (72.8) |
| Age, n acceptable (%) | 1577 (69.8) | 776 (70.3) |
| Risk calculator, n acceptable (%) | 1499 (66.4) | 722 (65.3) |
| Only men (among men), n acceptable (%) | 770 (68.2) | 392 (72.2) |
| Only men (among women), n acceptable (%) | 459 (40.7) | 229 (41.0) |
| Medical file, n willing to disclose (%) | 1802 (79.8) | 884 (80.0) |
| Waist circumference, n willing to disclose (%) | 1855 (82.2) | 909 (82.4) |
| Smoking status, n willing to disclose (%) | 2028 (89.9) | 992 (89.9) |
| Family history, n willing to disclose (%) | 1965 (87.1) | 964 (87.3) |
| Blood sample, n willing to disclose (%) | 1984 (87.9) | 959 (86.9) |

**Supplemental Table S6.** Baseline characteristics subsample 2.

|  | **Overall** | | | **Transnasal endoscopy scenario** | | **Ingestible cell-collection device scenario** | | **Breath test scenario** | |
| --- | --- | --- | --- | --- | --- | --- | --- | --- | --- |
|  | **Original sample (N=2258)** | **Subsample 2 (N=683)** | **Dutch population** | **Original sample (N=539)** | **Subsample 2 (N=157)** | **Original sample (N=547)** | **Subsample 2 (N=173)** | **Original sample (N=1172)** | **Subsample 2 (N=353)** |
| 45 – 54, n (%) | 627 (27.8) | 110 (16.1) | 35.1 | 132 (24.5) | 28 (17.8) | 163 (29.8) | 24 (13.9) | 332 (28.3) | 58 (16.4) |
| 55 – 64, n (%) | 832 (36.8) | 311 (45.5) | 35.9 | 207 (38.4) | 66 (42.0) | 204 (37.3) | 88 (50.9) | 421 (35.9) | 157 (44.5) |
| 65 – 74, n (%) | 797 (35.3) | 260 (38.1) | 29.0 | 199 (36.9) | 62 (39.5) | 179 (32.7) | 60 (34.7) | 419 (35.8) | 138 (39.1) |
| Female, n (%) | 1127 (49.9) | 324 (47.4) | 50.3 | 265 (49.2) | 77 (49.0) | 267 (48.8) | 77 (44.5) | 595 (50.8) | 170 (48.2) |
| Education level, n (%) |  |  |  |  |  |  |  |  |  |
| Lower | 535 (23.7) | 157 (23.0) | 27.8 | 129 (23.9) | 36 (22.9) | 126 (23.0) | 38 (22.0) | 280 (23.9) | 83 (23.5) |
| Middle | 857 (38.0) | 264 (38.7) | 37.6 | 194 (36.0) | 56 (35.7) | 217 (39.7) | 71 (41.0) | 446 (38.1) | 137 (38.8) |
| Higher | 866 (38.4) | 262 (38.4) | 33.6 | 216 (40.1) | 65 (41.4) | 204 (37.3) | 64 (37.0) | 446 (38.1) | 133 (37.7) |
| Civil status, n married (%) | 1598 (70.8) | 469 (68.7) | 62.3 | 368 (68.3) | 104 (66.2) | 384 (70.2) | 114 (65.9) | 846 (72.2) | 251 (71.1) |
| Personal history of cancer, n yes (%) | 284 (12.6) | 92 (13.5) | 7.2 | 70 (13.0) | 22 (14.0) | 69 (12.6) | 20 (11.6) | 145 (12.4) | 50 (14.2) |
| Participated in other cancer screening, n yes (%) | 1726 (88.2)^a^ | 451 (66.0)^b^ | 67.6 | 427 (90.1) | 106 (67.5) | 398 (82.0) | 106 (61.3) | 901 (89.2) | 239 (67.7) |

^a^ Among individuals eligible for any program (n= 1958).
^b^ Among individuals eligible for any program (n= 683).

**Supplemental Table S7.** Anticipated discomfort, acceptability test performance, and intended uptake of screening scenarios in subsample 2.

|  | **Upper endoscopy scenario** | | **Transnasal endoscopy scenario** | | **Ingestible cell-collection device scenario** | | **Breath test scenario** | |
| --- | --- | --- | --- | --- | --- | --- | --- | --- |
|  | **Original sample (N = 2258)** | **Subsample 2 (N=683)** | **Original sample (N = 539)** | **Subsample 2 (N=157)** | **Original sample (N = 547)** | **Subsample 2 (N=173)** | **Original sample  (N = 1172)** | **Subsample 2 (N=353)** |
| Anticipated discomfort, m (SD) | 5.1 (1.6) | 5.1 (1.6) | 4.7 (1.5) | 4.5 (1.4) | 4.0 (1.6) | 4.0 (1.6) | 1.5 (0.9) | 1.5 (0.9) |
| Anticipated gagging, m (SD) | 5.1 (1.6) | 5.1 (1.6) | 4.1 (1.7) | 3.8 (1.6) | 4.2 (1.7) | 4.2 (1.8) | 1.5 (1.0) | 1.5 (1.0) |
| Acceptability test performance, m (SD) | n/a | n/a | 4.0 (1.5) | 4.1 (1.5) | 4.2 (1.4) | 4.4 (1.5) | 4.3 (1.5) | 4.3 (1.5) |
| Intended participation, n(%) |  |  |  |  |  |  |  |  |
| Yes, absolutely | 785 (34.8) | 220 (32.2) | 135 (25.0) | 37 (23.6) | 165 (30.2) | 46 (26.6) | 756 (64.5) | 209 (59.2) |
| Yes, I think so | 973 (43.1) | 285 (41.8) | 232 (43.0) | 64 (40.8) | 244 (44.6) | 77 (44.5) | 361 (30.8) | 118 (33.4) |
| No, I don’t think so | 411 (18.2) | 136 (19.9) | 131 (24.3) | 41 (26.1) | 112 (20.5) | 36 (20.8) | 36 (3.1) | 13 (3.7) |
| No, absolutely not | 60 (2.7) | 29 (4.2) | 27 (5.0) | 8 (5.1) | 8 (1.5) | 2 (1.2) | 5 (0.4) | 4 (1.1) |
| Missing | 29 (1.3) | 19 (2.8) | 14 (2.7) | 7 (4.5) | 18 (3.2) | 12 (6.9) | 14 (1.2) | 9 (2.5) |

**Supplemental Table S8.** Acceptability eligibility criteria subsample 2.

|  | **Original sample (N = 2258)** | **Subsample 2 (N=683)** |
| --- | --- | --- |
| GERD, n acceptable (%) | 1655 (73.3) | 474 (69.3) |
| Age, n acceptable (%) | 1577 (69.8) | 453 (66.3) |
| Risk calculator, n acceptable (%) | 1499 (66.4) | 449 (65.7) |
| Only men (among men), n acceptable (%) | 770 (68.2) | 225 (62.9) |
| Only men (among women), n acceptable (%) | 459 (40.7) | 141 (43.6) |
| Medical file, n willing to disclose (%) | 1802 (79.8) | 521 (76.3) |
| Waist circumference, n willing to disclose (%) | 1855 (82.2) | 541 (79.2) |
| Smoking status, n willing to disclose (%) | 2028 (89.9) | 595 (87.1) |
| Family history, n willing to disclose (%) | 1965 (87.1) | 578 (84.6) |
| Blood sample, n willing to provide (%) | 1984 (87.9) | 578 (84.6) |

**References**

1. Statistics Netherlands. (<https://opendata.cbs.nl/statline#/CBS/nl/> (Accessed 24 May 2023)).

2. Netherlands Cancer Registry (NCR) maintained by the Netherlands Comprehensive Cancer Organisation (IKNL). (<https://nkr-cijfers.iknl.nl/#/viewer/2cd1ea04-709d-43bc-9c79-cbe2710692f7> (accessed on 24 May 2023)).

3. Key Performance Indicators population screening programmes the Netherlands. (<https://www.bevolkingsonderzoeknederland.nl/en/bewezen-effectief/#home> (Accessed 24 May 2023)).
